# Systematic evaluation of colorectal cancer organoid system by single-cell RNA-Seq analysis

**DOI:** 10.1186/s13059-022-02673-3

**Published:** 2022-04-28

**Authors:** Rui Wang, Yunuo Mao, Wendong Wang, Xin Zhou, Wei Wang, Shuai Gao, Jingyun Li, Lu Wen, Wei Fu, Fuchou Tang

**Affiliations:** 1 Department of General Surgery, School of Life Sciences, Biomedical Pioneering Innovation Center, Third Hospital, Peking University, Beijing, 100871 People’s Republic of China; 2 Beijing Advanced Innovation Center for Genomics & Ministry of Education Key Laboratory of Cell Proliferation and Differentiation, Beijing, 100871 People’s Republic of China; 3 Peking-Tsinghua Center for Life Sciences, Peking University, Beijing, 100871 People’s Republic of China; 4 Peking University Third Hospital Cancer Center, Beijing, 100191 People’s Republic of China; 5 Present address: Department of Breast Surgery, The First Affiliated Hospital of Zhengzhou University, Zhengzhou, Henan 450052 People’s Republic of China

## Abstract

**Background:**

Patient-derived organoid culture is a powerful system for studying the molecular mechanisms of cancers, especially colorectal cancer (CRC), one of the most prevalent cancers worldwide. There are two main types of 3D culture methods for colonic cells, but the similarities and differences between gene expression patterns in different culture media remain largely unexplored.

**Results:**

Here, we establish patient-derived organoids from colorectal cancer patients and perform single-cell RNA-Seq for pairwise samples from seven patients for both organoids and their corresponding tumor and normal tissues in vivo. We find that organoids derived from tumor tissues faithfully recapitulate the main gene expression signatures of cancer cells in vivo. On the other hand, organoids derived from normal tissues exhibited some tumor-like features at the whole transcriptome level but retained normal genomic features, such as CNVs, point mutations, and normal global DNA methylation levels, for both cultural media. More importantly, we show that conditioned medium outperforms chemical-defined medium in long-term culture of tumor epithelial cells. Finally, we mutually exchange the culture medium for the organoids and find that after interchanging the medium, the organoid cells basically maintain the transcriptome characteristics of the original medium.

**Conclusions:**

Our work gives a thorough evaluation of both the cultural conditions and the biological features of organoids of CRC patients.

**Supplementary Information:**

The online version contains supplementary material available at 10.1186/s13059-022-02673-3.

## Background

Due to the highly heterogeneous nature of tumors, it is very challenging to study the molecular mechanisms of tumorigenesis. Cancer cell lines have been widely used as preclinical model systems for molecular studies of cancer and anticancer drug screening. However, due to the lack of the cell-cell interactions and three-dimensional (3D) tumor architecture, two-dimensional (2D) cultured cancer cell lines cannot faithfully represent and maintain the genetic diversities of in vivo tumors [1, 2]. Recently, in vitro-derived 3D organoid culture systems have been developed that may overcome many of these limitations. Tumor-derived organoids of several types of cancer, such as colorectal (CRC) [3, 4], breast [5, 6], bladder [7], lung [8, 9], pancreatic [10], and prostate cancer [11, 12], have now been successfully established and widely used.

## Results

**Fig. 1:**
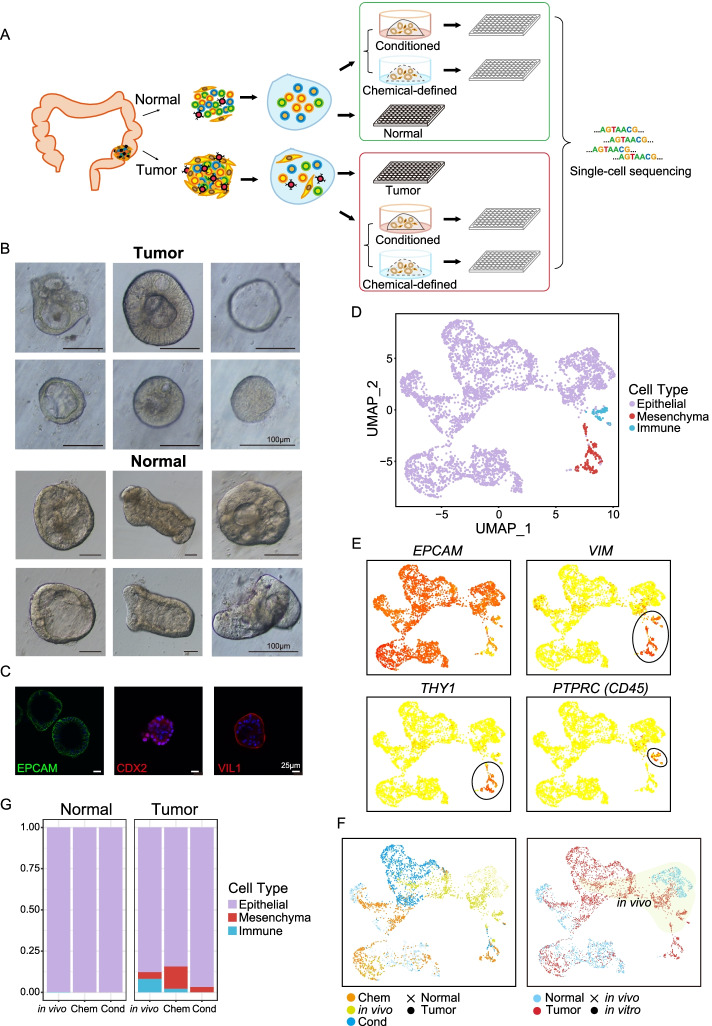
Global patterns of single-cell RNA-Seq profiles and cell type identification. **A** Schematic workflow of organoid culture and single-cell RNA-Seq data generation. **B** Zoomed-in bright-field images of tumor and paired adjacent normal-tissue-derived organoids. The tumor-derived organoids showed diverse morphologies. **C** Whole-mount immunofluorescent staining of intestine-specific markers on patient-derived organoids. EPCAM, epithelial cell marker; CDX2 and VIL1, intestinal epithelial cell markers. Basic information for these genes can be found in Additional file 4: Table S3. **D** tSNE visualization of single cells from tissues in vivo and organoid cultures, where individual points correspond to single cells. Cells are colored by annotated cell types. **E** Expression patterns of well-known cell-type markers were projected onto the tSNE map. The colors from yellow to red represent expression levels from low to high. Black circles highlight the position of immune cells and mesenchymal cells. EPCAM, epithelial cell marker; VIM and THY1, mesenchymal cell markers; PTPRC (CD45), immune cell marker. **F** tSNE clustering of cells and colors represent culture media or patients. **G** Cell type ratios for the in vivo tumor and adjacent normal tissues as well as the in vitro-derived organoids in different culture media. Colors represent different cell types

To fully evaluate the organoid culture system, we performed a high-precision single-cell RNA-Seq survey of 6225 single cells from tumors and adjacent normal tissues in vivo as well as corresponding patient-derived organoid samples that were cultured in two mainstream culture media from 7 patients with colorectal cancer to investigate their gene expression signatures (Fig. 1A and Additional file 1: Fig. S1A) [3, 11, 13]. We also applied whole-exome sequencing (WES), whole-genome sequencing (WGS), whole-genome bisulfite sequencing (PBAT), and Sanger sequencing to characterize their genomic and epigenomic features. We found that, overall, tumor-derived organoids in vitro faithfully maintained the gene expression signatures, gene regulatory networks, DNA methylation levels, and genomic mutations such as copy number variations (CNVs) and point mutations (SNVs), of tumor cells in vivo. However, the adjacent normal-tissue-derived organoids, despite retaining normal genomes, changed their gene expression profiles drastically and acquired some tumor-like gene expression signatures in vitro.

### Establishment and histopathological characterization of colorectal cancer organoids

Using a previously described organoid culture system, we successfully established a panel of organoid cultures derived from surgical resection specimens from patients with multiple types of CRC [3]. In detail, 7 tumor organoid cultures and 7 paired adjacent normal organoid cultures derived from seven CRC patients were successfully established and high-precision scRNA-Seq analysis was performed for them (Fig. 1A, Additional file 1: Fig. S1A and Additional file 2: Table S1). For 4 of the patients, their tumor and normal-derived organoids were cultured in both a chemical-defined medium and a conditioned medium (Fig. 1A and Additional file 1: Fig. S1A). Consistent with previous studies, we observed that organoids derived from tumor tissues presented more diverse morphologies than adjacent normal-derived organoids (Fig. 1B) [3]. As shown in Fig. 1B, the morphologies of tumor organoids ranged diversely from thin-walled cystic to compact structures. Then, we performed immunofluorescence staining of several epithelial cell makers (EPCAM, CDX2, and VIL1) for these cultured organoids (Fig. 1C, Additional file 1: Fig. S1B and Additional file 4: Table S3). The results showed that the organoids we cultured expressed well-known markers of intestinal epithelial cells as expected.

### Cell-type composition of colorectal cancer organoids

Previous colon organoid-related studies mainly analyzed the samples at the bulk levels, which cannot reveal cell-type diversity and heterogeneity [3, 14]. However, recent advances of single-cell RNA-Seq techniques provide us with an avenue to investigate the tumor composition and functional heterogeneity [15]. To explore the cellular diversity, we performed high-precision single-cell RNA-Seq for seven paired organoids derived from tumor and adjacent normal tissues in culture as well as the corresponding tumor and adjacent normal tissues in vivo from the same patients. That is, we dissociated the tumor and adjacent normal tissues from each patient into single-cell suspension and then used half of them for single-cell RNA-Seq analysis directly. For the remaining half of the sample, we cultured them to derive organoids. After the organoids were successfully established, we isolated organoid cells and performed a single-cell RNA-Seq analysis (Fig. 1A). In this way, we obtained single-cell transcriptome data for each patient’s tumor and adjacent normal tissues in vivo, as well as organoids derived from the tumor and adjacent normal tissues of the same patient. After quality control, the high-precision single-cell transcriptomes of 4445 cells were retained for further analyses. Based on their global gene expression patterns, we partitioned these cells into three clusters, which were annotated as epithelial cells, immune cells, and mesenchymal cells (Fig. 1D–F and Additional file 3: Table S2). We found that adjacent normal tissues in vivo and normal tissue-derived organoids were mainly composed of epithelial cells. Immune cells were strongly enriched in tumor tissues in vivo but were barely detected in tumor-derived organoids as expected (Fig. 1G). It is worth noting that chemical-defined medium may also promote the growth of mesenchymal cells in vitro, because they account for up to 13.5% of the cultured cells in a chemical-defined medium (Fig. 1E–G). In addition, we further analyzed epithelial subtypes and identified 7 subtypes in total (Additional file 1: Fig. S2A-B). Globally, we have found that compared with normal tissues or organoids derived from normal tissues, tumor tissues or tumor-derived organoids have more percentage of stem cell-like cells (Additional file 1: Fig. S2C). In addition, in vitro culture increases the ratio of stem cell-like cells in normal-derived organoids. It showed that the organoids were mainly composed of epithelial cells which implied that in vitro culture tended to enrich for epithelial cells, as previously reported [3].

### Adjacent normal-tissue-derived organoids exhibit some tumor-like features at the whole transcriptome level

**Fig. 2:**
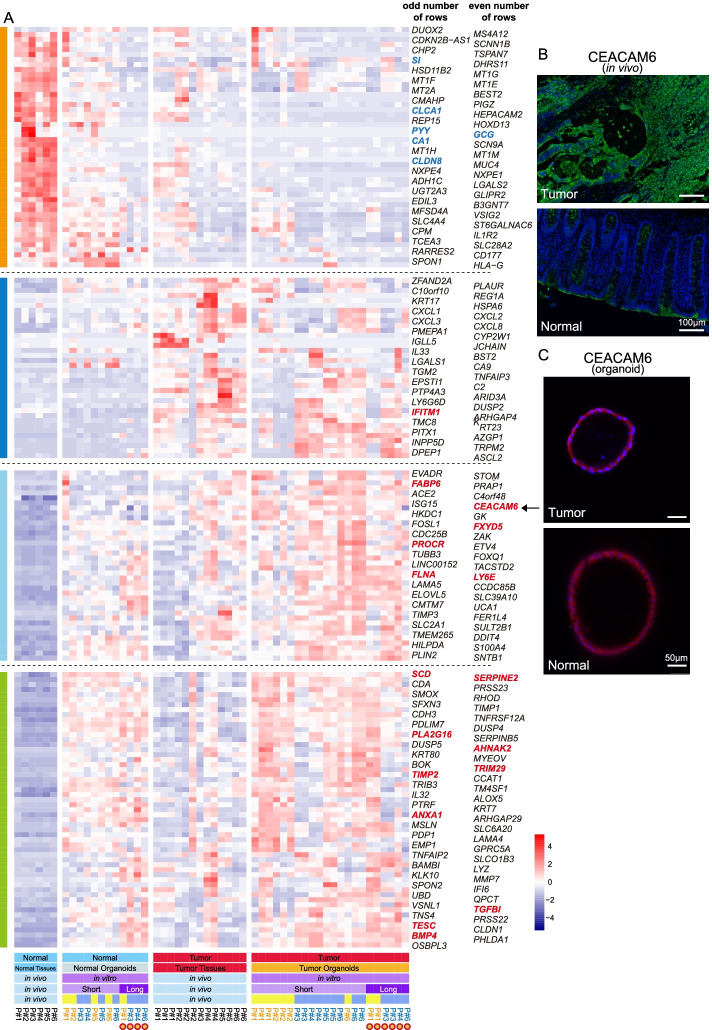
Normal tissue-derived organoids exhibited some tumor-like features at the transcriptome level. **A** Expression patterns of differentially expressed genes (DEGs) of in vivo tumor and adjacent normal tissues. Colors from blue to red represent low to high expression. Short, short-term culture; Long, long-term culture. Patient number in black, blue, and orange represent cells collected from in vivo tissues, in vitro chemical-defined medium, and conditioned medium. The detail description for some of these genes can be found in Additional file 4: Table S3. Many of tumor highly specifically expressed genes were reported to play important roles in colorectal cancer metastasis and progression (such as *TGFBI, SCD, TESC, CEACAM6*), while some of normal tissue highly expressed genes were well-known intestinal cell-type-specific marker genes, such as *SI*, *CA1,* and *PYY*. **B** Immunostaining of CEACAM6 in the adjacent normal and tumor tissues in vivo. Scale bar, 100 μm. **C** Immunostaining of CEACAM6 in the in vitro normal-tissue- and tumor-derived organoids. Scale bar, 50 μm

Next, we investigated whether patient-derived organoids could faithfully recapitulate the transcriptome features of the tumors and adjacent normal tissues in vivo. We extracted the differentially expressed genes (DEGs) between the tumor and normal tissues *in vivo* and analyzed their expression patterns in cultured organoids in vitro. It showed that several well-known intestinal marker genes (such as *CLCA1, PYY, GCG, CA1, CLDN8*, and *SI*) were highly expressed by both in vivo normal epithelial cells and in vitro normal tissue-derived organoid epithelial cells, which reflect that the normal tissue-derived organoids maintained the characteristic transcription features as well as cell type heterogeneities of in vivo intestinal tissue (Fig. 2A and Additional file 4: Table S3). As expected, tumor-derived organoids highly expressed in vivo tumor-specific genes, many of which have been widely reported to be closely related to colorectal cancer progression and metastasis, such as *PROCR*, *SCD*, *BMP4*, *CEACAM6*, *TESC*, and *TGFBI* (Fig. 2A and Additional file 4: Table S3). However, to our surprise, normal-tissue-derived organoids also highly expressed part of these in vivo tumor-specific genes (Fig. 2A). Moreover, we performed the immunofluorescence staining of the tumor-specific gene CEACAM6 for both in vivo tissue sections and organoids in culture (Fig. 2B, C). The results showed that CEACAM6 was highly expressed by only the tumor cells but not the adjacent normal tissues in vivo (Fig. 2B). However, both tumor and normal-tissue-derived organoids in culture highly expressed CEACAM6, verifying that normal-tissue-derived organoids acquired some gene expression features of colorectal cancer cells (Fig. 2C).

**Fig. 3:**
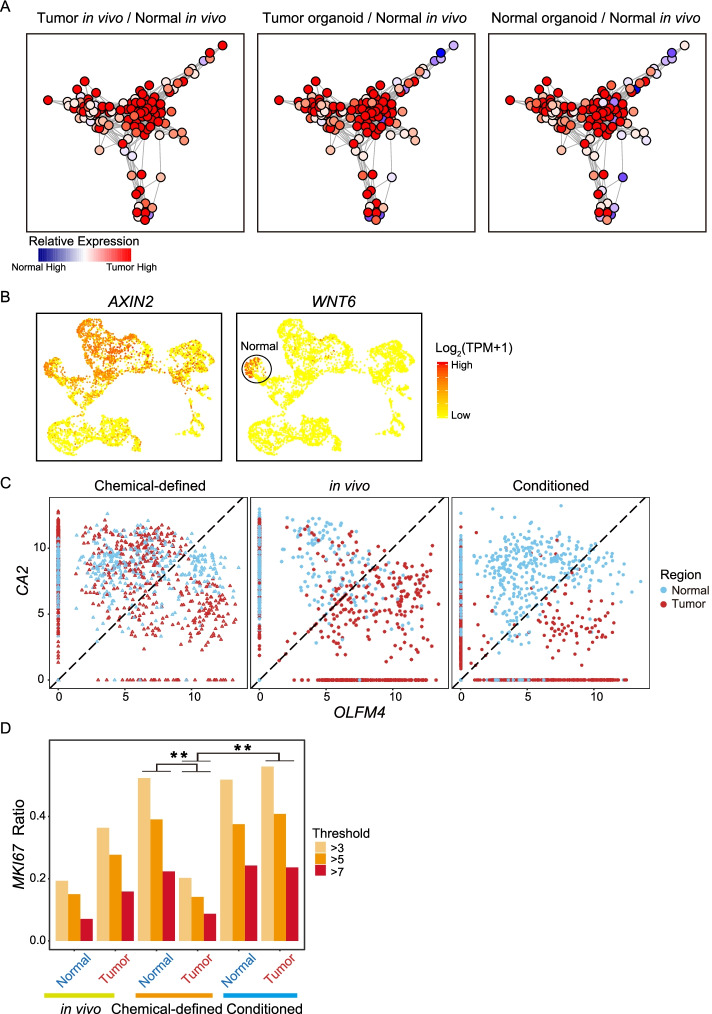
Cancer-derived organoids can maintain in vivo gene regulatory networks and comparisons of two different culture media. **A** In vivo tumor-specific gene regulatory network. Nodes represent tumor-specific genes and their regulatory genes. The colors represent the fold change in the mean gene expression levels between the two sources of epithelial cells. (Left: tumor cells in vivo compared with normal epithelial cells in vivo; Middle: tumor-derived organoid compared with normal epithelial cells in vivo; Right: normal-derived organoid compared with normal epithelial cells in vivo). **B** Expression patterns of Wnt signaling pathway target genes *AXIN2* and *WNT6* were projected onto the tSNE map. The colors from yellow to red represent expression levels from low to high. **C** Dot plot represents the expression levels of intestinal pluripotency marker *OLFM4* and enterocyte marker *CA2* for each cell. Colors represent sample regions. Chem, chemical-defined medium; Cond, conditioned medium. **D** Bar plot showing the ratio of cells that expressed different levels of proliferation marker *MKI67*. Colors represent different expression range of *MKI67*. ** indicates *p* value < 0.01

We wondered whether the gene regulatory network also exhibited similar patterns, so we explored the tumor-specific gene regulatory network among the in vivo and in vitro samples. The result shows that compared with normal epithelial cells in vivo, both normal tissue- and tumor-derived organoids showed in vivo cancer cell-specific gene regulatory networks (Fig. 3A). Taken together, these data revealed that at both the RNA, protein, and gene regulatory network levels, normal-tissue-derived organoids acquired some tumor-like features after culture in vitro.

### Transcriptomic feature comparisons between different cultural media

As shown in Fig. 1F, the cell type compositions were different between chemical-defined and conditioned media; thus, we further explored the differences of gene expressions between these two cultural media. Wnt signaling pathway plays vital roles in the self-renewal of many types of stem cells and malignant transformation, and we found that Wnt signaling target gene *AXIN2* is highly expressed by cells that were cultured in a conditioned medium (Fig. 3B and Additional file 1: Fig. S2D). In addition, another Wnt signaling target gene *WNT6* was mainly expressed by normal tissue-derived organoids that were cultured in a conditioned medium (Fig. 3B and Additional file 1: Fig. S2D). Next, we compared the expression of stem/progenitor cell marker gene (*OLFM4*) and differentiation maker gene (*CA2*) for epithelial cells in different cultural media and in vivo tissues (Fig. 3C and Additional file 1: Fig. S2B). It showed that organoid cells that were cultured in a conditioned medium maintained the differential expression patterns of *CA2* and *OLFM4* as those of the corresponding epithelial cells in vivo. In details, cancer cells in vivo and corresponding organoids cultured in a conditioned medium expressed higher levels of stem/progenitor cell maker gene *OLFM4*, while normal epithelial cells and corresponding organoids in a conditioned medium expressed higher levels of differentiation gene *CA2*. However, in a chemical-defined medium, tumor tissue-derived and normal tissue-derived organoid cells are mixed together, and there are no significant differences in the expression of *CA2* and *OLFM4*, suggesting that chemical-defined medium favors stem cell-like features of normal epithelial cells whereas disfavors differentiation/mature features of them (Fig. 3C).

Next, we measured the cell proliferation in different culture media through the expression of *MKI67*. In a chemical-defined medium, normal tissue-derived organoids had much faster cell proliferation than tumor-derived organoids. On the other hand, in a conditioned culture medium, normal tissue- and tumor tissue-derived organoids showed a comparable proliferation rate (Fig. 3D). This suggests that a chemical-defined medium favors the growth of normal epithelial cells whereas a conditioned medium in general showed no bias for the growth of normal epithelial cells and cancer epithelial cells.

### Lineage tracing with mitochondrial mutations

Recent research has reported that mutations in mitochondria DNA can be used as genetic markers to trace the tumor clonal structures [16]. To this end, we called the mitochondrial mutations for both in vivo epithelial cells and corresponding patient-derived organoids at single-cell levels. For patients #1 and #2, we picked single cells after dissociating a single organoid, so we know the individual organoid origin of the cells (Additional file 1: Fig. S3A). For other patients, organoids in the entire culture dish were dissociated first and then we picked the single cells for subsequent RNA sequencing analysis (Additional file 1: Fig. S3B).

**Fig. 4:**
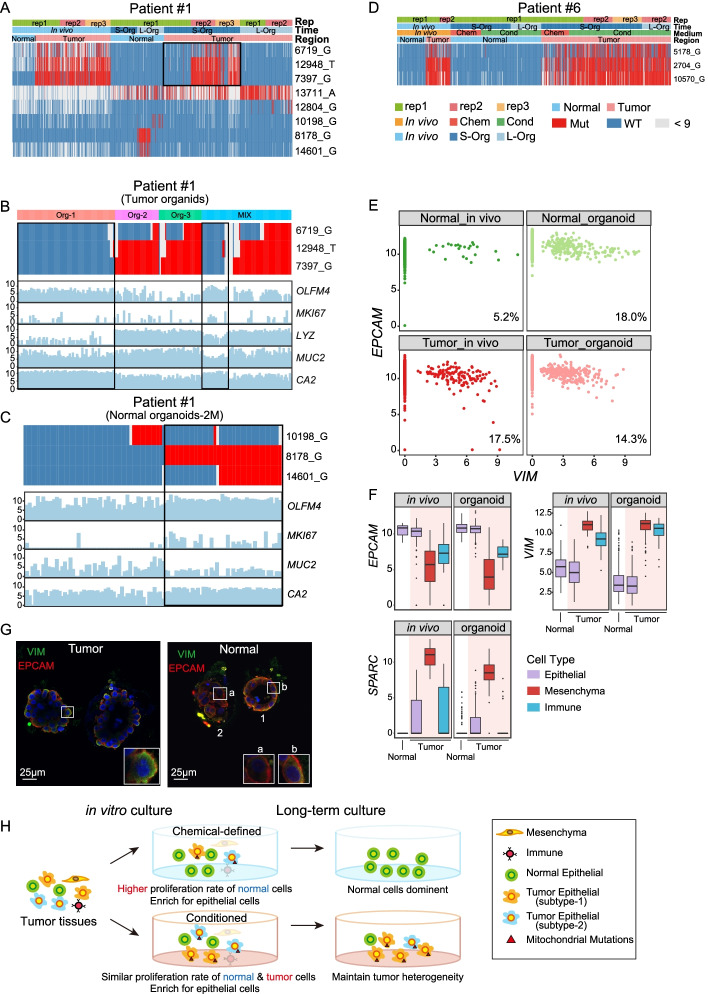
Cancer cell identification and lineage tracing with mitochondrial mutations. **A** Heatmap showing the mitochondrial mutations in cells of patient #1 that cultured in chemical-defined medium. Red represents mutant site, blue represents wild type, and gray represents site with sequence reads lower than 9. L-Org, long-term cultured organoids; S-Org, short-term cultured organoids. Chem, chemical-defined medium. Cond, conditioned medium. **B** Heatmap showing the mitochondrial mutations in tumor organoid cells of patient #1 that cultured in chemical-defined medium. Cells of three single organoid spheres and a mixture of several organoids were showed. Bar plot showing the corresponding expression levels of intestinal cell type markers. *OLFM4*, pluripotent cell marker; *MKI67*, cell proliferation marker; LYZ, Paneth cell marker; *MUC2*, goblet cell marker; *CA2*, enterocyte marker. **C** Heatmap showing the mitochondrial mutations in long-term-cultured normal organoid cells of patient #1 that cultured in chemical-defined medium. Bar plot showing the corresponding expression levels of intestinal cell type markers. **D** Heatmap showing the mitochondrial mutations in cells of patient #6 that cultured in both chemical-defined medium and conditioned medium. Red represents mutant site, blue represents wild type, and gray represents site with reads lower than 9. Chem, chemical-defined medium; Cond, conditioned medium. **E** Scatterplot showing the expression pattern of the epithelial cell marker *EPCAM* and classical mesenchymal cell marker *VIM* in each individual epithelial cell. The ratio of cells that co-expressed *EPCAM* and *VIM* is shown at the bottom right. **F** Box plot showing the expression levels of *EPCAM*, *SPARC,* and *VIM* in VIM-positive epithelial cells. The color of bar represents different cell types and the red background highlight cells in tumor. The black lines of box plot represent median values, the box limits indicate upper and lower quartiles, and the whiskers correspond to 1.5× the interquartile range. **G** Immunofluorescent staining of VIM and EPCAM on tumor- and normal-derived organoids. Three cells are shown at higher magnification at the lower right. Cell #a expressed only EPCAM, while Cell #b co-expressed VIM and EPCAM. **H** The schematic diagram summarizes the mitochondrial mutations and cell type changes of tumor cells in short-term and long-term cultures in two cultural media

Using mitochondrial mutations, we can accurately distinguish whether in vitro-cultured organoids are derived from cancer cells or normal epithelial cells at single-cell resolution. For example, we found that the majority of the cancer cells in vivo of patient #1 have tumor-specific mitochondria mutations on 6719_G, 12948_T, and 7397_G (Fig. 4A). All normal tissue-derived organoids of patient #1 are of wild-type at these three sites (Fig. 4A). For tumor-derived organoids, we picked cells from three individual organoids (Org-1, Org-2, Org-3, Additional file 1: Fig. S3A) and one batch of organoid mixtures (Mix, Additional file 1: Fig. S3B), some of the cells from Mix and all cells from Org-1 did not have tumor-specific mutations, whereas the majority of the cells from Org-2, Org-3 have tumor-specific mutations (Fig. 4B). This phenomenon may be explained by the presence of both cancer cells and normal epithelial cells in the in vivo tumor tissue, and both of them grew to form organoids in vitro. It is worth mentioning that in patient #1, the mitochondrial mutations were related to different epithelial cell types (Fig.4B, C). Epithelial cells with 6719_G, 12948_T, and 7397_G mutations highly expressed Paneth cell marker *LYZ*, while epithelial cells that were wild-type at corresponding positions highly expressed stem/progenitor cell marker *OLFM4* (Fig. 4B). A similar pattern was also observed in patient #2 (Additional file 1: Fig. S3C-D).

We have shown that the proliferation rate of cancer cells in a chemical-defined medium is much slower than that of normal epithelial cells (Fig. 3D). Therefore, we next compared the proportion of cancer cells in organoids cultured in different media. In patient #1 whose cells were cultured with chemical-defined medium, we found that long-term cultured tumor-derived organoids lost cells with tumor-specific mutations, suggesting that normal epithelial cells grew faster and cancer cells were gradually diluted by normal epithelial cells during in vitro culture (Fig. 4A, H). But for patient #6, long-term cultured organoid cells in a conditioned medium maintain tumor-specific mutations, suggesting that cancer cells are maintained as the major population of organoids cultured in a conditioned medium (Fig. 4D, H).

For patient #6, we used both chemical-defined and conditioned media to culture the normal tissue- and tumor tissue-derived organoid cells (Fig. 4D). We found that organoid cells cultured in both the chemical-defined medium and conditioned medium maintained the in vivo mitochondrial mutation features. However, organoids cultured in the chemical-defined medium for both tumor tissues and normal tissues showed decreased proliferation rate while those cultured in a conditioned medium better-maintained cell proliferation rate for a long time (Fig. 4D). As for patient #3 whose organoid cells were cultured in the conditioned medium for a long time, it revealed that organoids from both tumor tissues and normal tissues after long-term culture in the conditioned medium still maintained mitochondrial mutation patterns, indicating that the conditioned medium can maintain proliferation of both normal epithelial cells and cancer epithelial cells well (Additional file 1: Fig. S3E). Taken together, our result indicates that a conditioned medium is more suitable for epithelial cell growth and more suitable for long-term culture of both normal epithelial cells and cancer epithelial cells.

Epithelial-mesenchymal transition (EMT) often occurs during tumor progression, and it is reported that cancer cells would exhibit partial-EMT characteristics in order to enhance their invasive properties and generate circulating tumor cells and cancer stem cells. When cancer cells showed a partial-EMT state, it would simultaneously exhibit both mesenchymal and epithelial characteristics [17]. *VIM* is a widely used mesenchymal cell marker, while *EPCAM* is a well-known epithelial cell marker [18, 19]. Our data showed that normal tissue-derived organoids exhibited some tumor-like features, so we explored the expression patterns of *VIM* and *EPCAM* in the epithelial cells from all patients (Fig. 4E). We found that in vivo, just a small proportion of normal epithelial cells co-expressed *VIM* and *EPCAM*. However, tumor epithelial cells in vivo, as well as organoids derived from both normal tissues and tumor tissues all have higher proportions of epithelial cells co-expressing *VIM* and *EPCAM*, which were further verified at protein levels through immunofluorescence staining (Fig. 4G). This indicated that in vitro culture might favor the partial EMT features of tumor cells in organoids. To exclude the potential mesenchymal cell contamination during organoid culture, we further compared the expression levels of VIM between these VIM^+^ epithelial cells and mesenchymal cells. It showed that the expression levels of *VIM* in VIM^+^ epithelial cells are only half of that in mesenchymal cells (Fig. 4F). Therefore, we speculated that in vitro culture may promote EMT in organoids.

### Cultured organoids preserved the genomic and global DNA methylation features of corresponding tissues in vivo

Next, we checked the mutations and CNVs in these organoids and confirmed that the normal tissue-derived organoids contained no CNVs or tumor-specific point mutations. First, we inferred the CNVs by single-cell RNA-Seq data. It showed that all tumor tissue-derived organoids cultured in a conditioned medium have similar CNV patterns to the corresponding tumor samples in vivo (Fig. S4 and Fig. S8). However, part of tumor organoids cultured in a chemical-defined medium, especially after long-term culture lost CNVs, suggesting that the normal epithelial cells outperformed the cancer epithelial cells in a chemical-defined medium (Additional file 1: Fig. S4A). Moreover, through performing whole-genome sequencing and Sanger sequencing, we further verified that normal tissue-derived organoids remained normal at genomic levels (Additional file 1: Fig. S6 and Fig. S7A-B, Additional file 6: Table S4). It showed that only tumor-derived organoids exhibited CNVs and point mutations comparable to those of the corresponding tumor samples in vivo from the same patient (Additional file 1: Fig. S5 and Fig. S6).

In addition, we found that in vitro culture may enrich for specific subtypes of tumor epithelial cells in vivo. For example, in patient #1, we found that only a fraction of the cancer cells in vivo had *SMAD4* deletions, but essentially all tumor tissue-derived organoid cells from this patient had *SMAD4* deletions which were further verified by Sanger sequencing (Additional file 1: Fig. S7C). This suggested that compared to the *SMAD4* wild-type cancer cells, the culture condition favors more the growth of cancer cells with *SMAD4* deletion. Similar patterns were also observed in patient #8, where cancer cells with strong CNVs were enriched after culture in vitro (Additional file 1: Fig. S7B).

Next, we investigated the mutation frequencies of tumor tissue-derived organoids in different culture media (Additional file 1: Fig. S5). Consistent with the results of mitochondrial mutations, the frequency of in vivo tumor-specific mutations in tumor tissue-derived organoids that are grown in a chemical-defined medium is reduced relative to corresponding tumor cells in vivo, which reflects the general decrease of the proportion of cancer cells in tumor-derived organoids cultured in chemical-defined medium. In contrast, the frequency of in vivo tumor-specific mutations in tumor tissue-derived organoids cultured in the conditioned medium is maintained or even increased.

In addition to studying CNVs and mutations, we also investigated the global DNA methylation levels of in vivo tissues and organoids that were cultured in a conditioned medium (Additional file 1: Fig. S7D-E). It showed that organoids could maintain global DNA methylation levels of corresponding tissues in vivo. In three of the patients we investigated, tumor tissues in vivo and tumor tissue-derived organoids have lower DNA methylation levels compared with normal tissues and normal tissue-derived organoids. Overall, these results indicated that cultured organoids can preserve the genomic and global DNA methylation levels of the tissues in vivo, including CNVs, point mutations, and DNA methylation patterns. Due to our shallow sequencing depth and small sample size, we did not compare DNA methylation differences across different regulatory elements, but only global DNA methylation patterns. Therefore, there may be some methylation differences at specific genes or regulatory elements that require further analysis using additional datasets.

### The exchange of culture medium of organoids

**Fig. 5:**
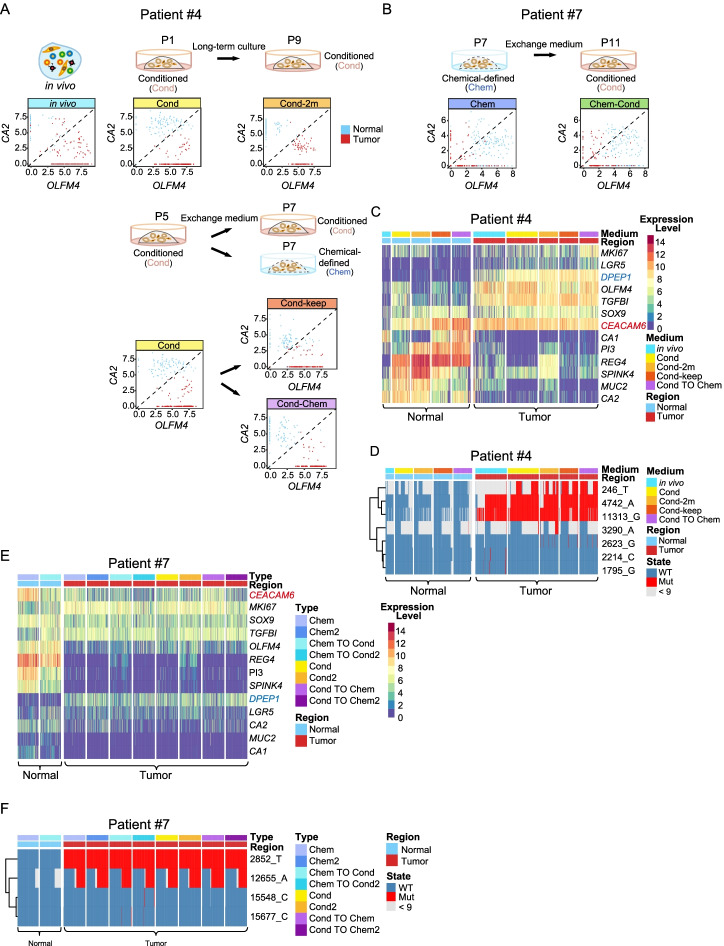
Culture medium exchange does not influence *CA2* and *OLFM4* expression patterns. **A** Schematic workflow of culture medium exchange for patient #4. Scatterplot showing the expression pattern of the enterocyte marker *CA2* and pluripotency cell marker *OLFM4* in each individual cell. Organoid cells of patient #4 were established in conditioned medium, after culture for 5 passages (P5), transfer to chemical-defined medium, or still stay in conditioned medium for 9 days and P7 cells were collected and performed scRNA-seq. “P” stands for passage. **B** Schematic workflow of culture medium exchange for patient #7. Scatterplot showing the expression pattern of the enterocyte marker *CA2* and pluripotency cell marker *OLFM4* in each individual cell. Organoid cells of patient #7 were established in chemical-defined medium, and P4 cells were transferred to conditioned medium cultured for 25 days and P11 cells were collected and performed scRNA-seq. “P” stands for passage. **C** Heatmap showing the expression patterns of selected genes in patient #4*.* In vivo tumor-specific genes: *DPEP1*, *CEACAM6*, and *TGFBI*. Intestinal subtype specific genes: *LGR5, SOX9, MKI67, OLFM4, CA1, REG4, SPINK4, MUC2*, and *CA2*. Colors from blue to red represent low to high expression levels. **D** Heatmap showing mitochondrial mutations of patient #4. Red represents mutant site, blue represents wild type, and gray represents site with reads lower than 9. Chem, chemical-defined medium; Cond, conditioned medium. **E** Heatmap showing the expression patterns of selected genes in patient #7. Colors from blue to red represent low to high expression levels. **F** Heatmap showing mitochondrial mutation of patient #7. Red represents mutant site, blue represents wild type, and gray represents site with reads lower than 9. Chem, chemical-defined medium; Cond, conditioned medium

To further verify that the differences in cell proliferation and maintenance of stem/progenitor cell features were caused by different culture media, we exchanged the culture medium of the organoids in subsequent experiments and performed high-precision single-cell RNA-Seq on these cells side by side (Fig. 5 and Additional file 1: Fig. S8). It showed that the organoid cells maintained the expression patterns of *OLFM4* and *CA2* in the original cultured medium after changing to the other culture medium (Fig. 5A-B). For example, organoid cells of patient #4 were initially cultured in the conditioned medium and tumor cells highly expressed *OLFM4* whereas the normal epithelial cells highly expressed *CA2*. After organoid cells were transferred to a chemical-defined medium, tumor cells, and normal epithelial cells still maintained this distinct expression patterns of *OLFM4* and *CA2* (Fig. 5A). On the other hand, organoid cells of patient #7 were initially cultured in a chemical-defined medium, and both tumor cells and normal epithelial cells showed similar expression levels of *OLFM4* and *CA2*. After the organoid cells were transferred into conditioned medium, this expression pattern of *OLFM4* and *CA2* was still maintained (Fig. 5B). We also analyzed the expression patterns of other signature genes in organoid cells of these two patients and showed that normal tissue-derived organoid cells highly expressed *REG4* and *SPINK4*, while tumor tissue-derived organoid cells highly expressed *DPEP1*. Similar to previous results, both normal tissue-derived organoids and tumor tissue-derived organoids highly expressed in vivo tumor-specific marker gene *CEACAM6* (Fig. 5C and E). In addition, the exchange culture medium did not influence the genomic features of the epithelial cells (Fig. 5D, F and Additional file 1: Fig. S8).

## Discussion

For decades, preclinical cancer research was mainly based on 2D-cultured cancer cell lines, which cannot faithfully represent the heterogeneous features of in vivo cancer cells due to unable to simulate the microenvironment of the original tumors, which grow three-dimensionally (3D). Patient-derived xenograft (PDX) models, as another useful tool for tumor study, can maintain cell-cell interactions in a 3D environment and capture tumor heterogeneity, but they are very time-consuming and difficult to utilize for high-throughput screenings [1, 2]. The recently developed organoid system can overcome many of these difficulties and serve as a suitable preclinical model for the evaluation of high-throughput drug response screening and the mechanistic studies of tumorigenesis, invasiveness, and metastasis [3, 4, 14].

By constructing patient-derived normal and tumor organoids with both a chemical-defined medium and conditioned medium, we found that the chemical-defined medium is more conducive to the proliferation of normal epithelial cells in vitro, while tumor cells and normal epithelial cells have comparable proliferation rates in the conditioned medium. Therefore, after long-term culture, tumor organoid cells will likely be totally replaced by normal epithelial cells from the original tumor tissues, and thus, tumor cells will be gradually lost in a chemical-defined medium. In addition, our data suggested that a conditioned medium can better reflect the differences of the degree of differentiation between tumor cells and normal epithelial cells in vivo than a chemical-defined medium. Similar to the corresponding epithelial cells in vivo, the tumor tissue-derived organoid cells in the conditioned medium showed high expression of stem/progenitor cell marker *OLFM4* and low expression of differentiation marker *CA2*, while normal tissue-derived organoid cells showed low expression of *OLFM4* and high expression of *CA2*. However, tumor tissue-derived organoid cells and normal tissue-derived organoid cells cultured in a chemical-defined medium have similar expression patterns of *CA2* and *OLFM4*, which cannot accurately mimic the expression patterns of the corresponding cells in vivo. In addition, similar to previous studies, we found that the success rate of using a conditioned medium to derive organoids is higher than that of the chemical-defined medium, especially for normal tissue-derived organoids. However, since the conditioned medium involved the cultivation of cell lines, the concentration of the components of the medium might vary in different batches and may have potential batch effects.

Next, by culturing the organoids in one medium for a period of time and then transferring them to another medium for further culturing, the expression of *OLFM4* and *CA2* in tumor tissue-derived organoid cells and normal tissue-derived organoid cells remained basically unchanged, indicating that the cultured medium that was initially used to derive the organoids plays a very critical role in the gene expression profiles of the organoids. However, the medium exchange experiments are only conducted on the organoids of two patients, and we did not measure the influence of a very long culture time after the medium exchange on the transcriptome changes. The main limiting factor is that it is difficult to culture organoids, especially for normal tissue-derived organoids, in the chemical-defined medium for a relatively long time. Therefore, we cannot obtain both normal and tumor organoid cells of the same patient that were cultured in two different media for a very long time.

Furthermore, it showed that both culture media can maintain the genomic, epigenomic, and transcriptomic characteristics of the tumor cells in vivo. Notably, our result showed that normal tissue-derived organoids exhibit some tumor-like features at the transcriptome levels, which were verified through immunofluorescent staining. However, the genomic and epigenomic features, such as CNVs, point mutations, and DNA methylation patterns, of normal-tissue-derived organoids still remain normal. This result suggested that normal-tissue-derived organoids need to be used with caution when studying gene expression features of normal epithelial cells in vivo. This suggests that when using organoids to study normal colonic cells, it is necessary to consider the influence of in vitro culture system on gene expression patterns.

## Conclusion

In summary, our single-cell RNA sequencing survey of tissues in vivo and corresponding organoids cultured in two different media, combined with genome and DNA methylome sequencing data, systematically evaluated the faithfulness of organoid culture systems for analyzing the molecular features of tumorigenesis of colorectal cancer. We showed that conditioned medium outperformed chemical-defined medium in long-term culture as well as maintenance of genomic, epigenomic, and transcriptomic features of both cancer cells and normal epithelial cells. We also showed that tumor-derived organoids in culture can faithfully reflect the gene expression patterns, gene regulatory networks, point mutations, CNVs, and DNA methylation patterns of tumor cells in vivo. On the other hand, normal-tissue-derived organoids maintain normal genomic features of in vivo normal epithelial cells but exhibit some tumor-like features at the transcriptome level.

## Methods

### Sample collection

Matched normal mucosa and primary tumor tissue were obtained from colorectal cancer patients from General Surgery, Peking University Third Hospital. From the freshly resected colon samples, both tumor and adjacent normal tissues were isolated and washed gently with cold 1X DPBS for at least five times and for 5 min each time. Then, the isolated tissues were cut into pieces with surgical scissors and digested with 2.5 mg/ml collagenase (type II and type IV; Invitrogen) to obtain a single-cell suspension. By applying a mouth pipette, we isolated single cells in good condition and placed them in a single-cell lysis mix for scRNA-Seq or scWGS-Seq. Normal tissues were taken from sites at least 10 cm away from the nearest tumor margin.

### Tissue dissociation and organoid culture

The organoid culture was performed as previously described [3], with minor modifications to the tissue dissociation protocol to improve cell viability. Specifically, well-washed tumor-adjacent normal tissues were carefully cut into 1–3-mm-long fragments by a scalpel. The muscle layer was then stripped off from the mucosa layer under a dissecting microscope. Subsequently, fragments were incubated in 5μM EDTA for 15 min with vigorously shaking. The isolated crypts were collected and further washed twice with the basal medium. Then, the crypts were allowed to mix with BME (Cultrex Reduced Growth Factor Basement Membrane Extract, Type 2, Bio-techne) and seeded in 24-well plates (50 μL BME/well). Culture medium was added after the BME was well solidified (approximately 20 min after seeding). Tumor tissues were then cut into small pieces and digested by collagenase (type II and type IV; Invitrogen) for 30 min at 37 °C with vigorous pipetting every 5 min. After digestion, dissociated tissues were passed through a 40-μm cell strainer (Corning). The cell suspension was first centrifuged at 400 g for 5 min at 4 °C and then was re-suspended with BME and seeded in 24-well plates (50 μL BME/well). The composition of the organoid culture medium was as follows:

#### Chemical-defined medium

The chemical-defined medium are advanced DMEM/F12 (Gibco), 100 U/mL penicillin/streptomycin (Gibco), Primocin (InvivoGen), 2 mM GlutaMAX (Gibco), 0.5 μM A83-01 (Tocris), 1x B27 (Gibco), 5 μM SB202190 (Sigma), 100 nM prostaglandin E2 (Tocris), 0.5 μg/mL R-spondin (Peprotech), 4mM nicotinamide (Sigma), 10 nM gastrin I (Sigma), 50 ng/ml EGF (Peprotech), 100 ng/ml Noggin (Peprotech), 100 ng/mL WNT3A (Millipore), 10ng/ml FGF-10 (Peprotech), and 10ng/ml FGF-basic (Peprotech). Notably, 10 μM Y-27632(Selleck) was supplemented to the medium in the first week and the medium was changed every 2 days.

#### Conditioned medium

Conditioned medium are 50% conditioned media (1:1 diluted with Advanced DMEM/F12), 100 U/mL penicillin/streptomycin, Primocin (InvivoGen), 2 mM GlutaMAX (Gibco), 0.5 μM A83-01 (Tocris), 1x B27 (Gibco), 5 μM SB202190 (Sigma), 100 nM prostaglandin E2 (Tocris), 4mM nicotinamide (Sigma), 10 nM gastrin I (Sigma), 50 ng/mL EGF (Peprotech), 10ng/mL FGF-10 (Peprotech), and 10ng/ml FGF-basic (Peprotech). The conditioned medium was prepared according to the protocol as previously described [13].

### Whole-mount immunofluorescence

The organoids were fixed in 4% paraformaldehyde at room temperature for 1 h, permeabilized in permeabilization solution at room temperature for 30 min, and then incubated in blocking solution at 4 °C overnight. The organoids were incubated with primary antibodies diluted 1:200 in blocking solution at 4 °C overnight and then washed three times for 10 min. The organoids were incubated with fluorescence-conjugated secondary antibodies and DAPI diluted at 1:500 in a blocking solution at 4 °C overnight. The organoids were transferred into a 3.5-cm dish, and mineral oil was used to cover the organoids to prevent liquid evaporation. The immunofluorescence was imaged using a Nikon A1RSi+ confocal microscope. The permeabilization solution was 1× DPBS supplemented with 1% Triton X-100, 1% BSA, and 1% goat serum. The blocking solution was 1× DPBS supplemented with 1% Triton X-100, 1% DMSO, 1% BSA, and 10% goat serum.

### Single-cell RNA-Seq library preparation and sequencing

Single-cell RNA-Seq sequencing libraries were generated based on the single-cell tagged reverse transcription (STRT) technique with a few modifications as described [20–22]. After enzymatic digestion, single cells were manually randomly picked into lysis buffer by mouth pipet and reverse transcribed. The primer involved was designed with unique molecular identifiers (UMIs) to eliminate duplication, cell-specific barcodes to label cells, and 25 nt oligo (dT) to capture target mRNA. Next, the second-strand cDNA was synthesized and followed by 19 cycles of PCR amplification. Approximately, 30–40 ng of purified DNA products was further amplified to introduce biotin tags and index sequences, which were then acoustically sheared into 300-bp fragments through the Covaris S2 system. Dynabeads MyOne Streptavidin C1 beads (Thermo Fisher), which capture cDNA fragments with biotin modified were then used. The libraries were constructed using a by Kapa Hyper Prep Kit (Kapa Biosystems) and were sequenced as paired-end 150-base reads on an Illumina HiSeq 4000 instrument.

### Whole-exome sequencing library construction

Genomic DNA was extracted using the DNeasy Blood & Tissue Kit (Qiagen). Approximately, 200 ng of DNA was sheared into fragments of 150–200 base pairs in length using the Covaris S2 system. After purification, the SureSelect Human All Exon V6 kit (Agilent) was applied for the construction of sequencing libraries following the provided manufacturer instructions. The libraries constructed were sequenced with the 150-bp paired-end model on the Illumina HiSeq 4000 platform.

### Single-cell whole-genome sequencing

Single-cell WGS was performed using the Multiple Annealing and Looping-Based Amplification Cycles (MALBAC) method [23]. The dissociated single cells were manually picked into 200 μL PCR tubes containing lysis buffer that was prepared in advance. The lysis of individual cells was carried out following 3 h of incubation at 50 °C and 30 min of protease inactivation at 70 °C. The genomic DNA was then directly processed for quasilinear preamplification and 17 cycles of exponential amplification. The libraries were constructed either in the same way as the scRNA-Seq libraries or using the TruePrepTM DNA Library Prep Kit V2 for Illumina® (Vazyme Biotech), both were sequenced on HiSeq 4000 platform and 150-bp paired-end reads were obtained for further analysis.

### Bulk whole-genome sequencing and RNA sequencing

The DNeasy Blood & Tissue Kit (Qiagen) was used to obtain genomic DNA. An average of 50 ng of DNA was used to construct WGS libraries following the TruePrepTM DNA Library Prep Kit V2 (Vazyme Biotech) manufacturer’s instructions. Population cell RNA was extracted using the RNeasy Mini Kit (Qiagen). After mRNA reverse transcription and cDNA amplification, approximately, 50 ng of DNA was used to perform library construction. Following the instruction that the TruePrepTM DNA Library Prep Kit V2 (Vazyme Biotech) provided, the prepared libraries were sequenced on an Illumina HiSeq 4000 system.

### Bulk DNA methylome sequencing library construction

The bulk DNA methylome library preparation was performed according to the previously reported method [24]. Briefly, genomic DNA was first extracted by the DNeasy Blood & Tissue Kit (Qiagen). Then, bisulfite conversion was performed with the Zymo^TM^ EZ-96 DNA Methylation-Direct MagPrep kit. Next, converted DNA was complemented by biotinylated random primers after beads-based purification. After capturing first-strand DNA by streptavidin beads, the second-strand DNA was synthesized with general random primers. Finally, PCR amplification and library purification were conducted to generate the final bulk DNA methylation library.

Antibodies used for immunofluorescence

**Table:** 

Antibody	Source	Identifier	Dilution ratio
EPCAM	Abcam	ab71916	1:200
CDX2	Abcam	ab76541	1:200
Villin (VIL1)	Abcam	ab201989	1:200
CEACAM6	Abcam	ab134074	1:200
MKI67	Abcam	ab15580	1:200
SOX9	Abcam	ab185966	1:200
Vimentin (VIM)	Abcam	ab8978	1:200

### Single-cell RNA sequencing data analysis

The paired-end sequencing data were processed as previously reported to get an expression matrix [25]. In detail, paired-end reads were first split according to the cell barcodes located in read 2 (R2). In the meantime, the 8bp UMIs located in read 2 was also pasted to the end of sequence read’s name. Next, low-quality reads and adapter contaminated reads were removed during the quality control pipeline. In detail, for each read R1, the polyA sequences were trimmed, followed by filtering for high-quality reads with the following criteria: (1) at least 40bp long, (2) more than half of the bases showing the sequence quality scores greater than 38, and (3) less than 10% of the bases showing N. For each read R2, the first 0–8bp and 9–16bp were extracted as cell barcode and UMI sequence and added to the read ID. The detailed codes have already been uploaded to the GitHub (https://github.com/WRui/Post_Implantation/blob/master/scRNA_UMI/Split_Barcodes/s01.Barcode_UMI_QC_per1w_V2.pl) [26]. Then, the clean reads were mapped to the human genome (hg19) using TopHat (version 2.0.14) and only uniquely mapped reads were kept for subsequent analyses [27]. The abundance of the transcripts was counted by HTSeq and reads with duplicated UMIs for each gene were excluded [28]. Finally, the expression matrix was normalized into TPM. Codes that were used for barcode split, quality control, reads mapping, and expression matrix acquisition can be found in the GitHub (https://github.com/WRui/Post_Implantation/tree/master/scRNA_UMI/) [26]. In total, we sequenced 6143 single cells. After filtering by detected gene number (>2000 genes) and cell-to-cell correlation (top 2 correlation higher than 0.6), 4445 cells were used for downstream analysis. The median ratio of reads that mapped to mitochondria is 13.4%, which indicates the relatively high quality of our data. In our manuscript, we used mutations in mitochondria to infer the clonal structure of organoids and in vivo tumor cells, and a little higher ratio of mitochondrial reads can make the clonal identification more accurate. Therefore, we do not use the mitochondrial ratio as a filter condition, but use the correlation coefficient between cells to filter out the outlier cells.

### Cell type identification

Seurat package was used to cluster the cells by performing RunPCA and RunUMAP functions with parameter dims=1:7. Then cell clusters were annotated as known cell types by checking known cell type-specific markers. In total, we identified three major cell types, epithelial cells (*EPCAM*), mesenchymal cells (*THY*), and immune cells (*PTPRC*). Since epithelial cells were the focus of our study, we further classified epithelial cells into four subtypes. Specifically, *MUC2* (Goblet), *CA2* (Enterocyte), and *FABP1*(Enterocyte) were used to annotate differentiated cell types, whereas the cells with high expression of *LGR5, MKI67*, and *OLFM4* were annotated as stem-like cell types.

### DEG identification

To identify cluster-specific expressed genes, the function Find_All_Markers with parameters thresh.use=2 in the Seurat R package was used [29].

### Correlation network construction

The in vivo tumor highly expressed gene correlation network was constructed according to a previously reported article [30]. First, in vivo tumor-specific genes were identified by Seurat package “FindMarkers” function with setting parameters as “ident.1=”Epithelial_Tumor_Invivo”,ident.2=”Epithelial_Normal_Invivo”,logfc.threshold=1.5,min.diff.pct = 0.25,min.pct = 0.25”. Then, we calculated the pairwise correlation matrix of all in vivo tumor cells by using in vivo tumor-specific genes described above. Then, a weighted adjacency network graph was generated through the performing graph.adjacency() function in igraph package and vertices with less than three edges were removed. Finally, the relative expression levels of two different conditions were mapped onto the network by using different colors. The gene list that is used to construct the network can be found in Additional file 4: Table S3.

### CNV calling using single-cell RNA-seq data

The CNV inferences with single-cell transcriptome data were performed according to a previously published method which was modified from inferCNV software [31]. Only genes with an average relative expression of more than 1.5 in all quality control passed single cells were used for CNV inference. First, a gene CNV score was defined as the average expression level of its neighboring 100 genes. Then, by subtracting the average CNV scores of all cells, the CNV scores are concentrated to zero. Finally, the relative CNV score was calculated by averaging the CNV scores of all genes in the 10M CNV window.

### Mitochondrial mutation calling using single-cell RNA-Seq data

We used TopHat output files for subsequent mitochondrial SNP calling using the GATK toolkit according to the online suggested SNP calling pipeline for RNA-Seq data. The detailed codes used for mitochondrial SNP calling cat to be found at GitHub (https://github.com/WRui/Metastatic-Colorectal-Cancer/blob/master/s02.RNA_SNP_Tang_chrM.sh).

### Whole-genome sequencing data analysis

Low-quality and index contaminate reads were first removed from the raw paired-end sequence data. Then, the clean reads were mapped to the human genome (hg19) with BWA (version 0.7.5a) and the duplicate reads were marked by Picard tools (version 1.130). Control-FREEC (v11.3) was used to detect copy-number changes. Based on the tutorial (http://boevalab.inf.ethz.ch/FREEC/tutorial.html#CONFIG), the config file was created, which used the following setting parameters: chrLenFile=/Path/To/hg19.genome, ploidy = 2, window = 10000000, chrFiles=/Path/to/hg19_chrSeq, maxThreads= 2, BedGraphOutPut = True, samtools = /path/to/samtools, outputdir = /path/to/outdir, inputFormat = BAM, mateOrientation =0, and mateFile = /path/to/bamfile. Then, we run “freec --conf config_file” to get a number of reads within per window (https://github.com/WRui/Colon_FAP/blob/master/04.FreeC_Work_Human.sh). Finally, we used R to normalize the total read depth and then use the “ggplot2” package to visualize the CNV patterns. The whole genome was divided into 10 M windows, and the total sequence reads were calculated for each window. For each window, the reads were first normalized by the total read depth of each sample and then normalized by the average read depth of all samples. The R script used to normalize read depth and data visualization can be found in the GitHub website (https://github.com/WRui/Colon_FAP/06.Plot_CNV.sh).

### Whole-exome sequencing data analysis

Duplicate marked mapping reads were obtained via WGS analysis, then GATK (Genome Analysis Toolkit, Version 3.8) and MuTect2 were used to call the somatic mutation [32, 33]. The whole pipeline can be separated into four steps: (1) local realignment around indels by RealignerTargetCreator and IndelRealigner, (2) base quality score recalibration with BaseRecalibrator and PrintReads, (3) somatic variants calling by MuTect2 with normal tissues as a control, and (4) variant annotation with SnpEff. The detailed codes and parameters setting for each step can be found in the GitHub website (https://github.com/WRui/Metastatic-Colorectal-Cancer) [25, 34].

### PBAT DNA methylome sequencing data analysis

First, the software trim_galore was used to remove adaptor sequences and low-quality reads with parameters: --quality 20 –stringency 3 –length 50 –clip_R1 9 –clip_R2 9 –paired –trim 1 –phred33. Then, clean reads were mapped to human genome hg19 using Bismark using paired-end mode with parameters: --fastq –non_directional [35]. Next, to improve data utilization, unmapped reads were remapped to the human genome with single-end mode. Only unique mapping reads were kept subsequent analysis. PCR duplicates were then removed by performing “samtools rmdup”.

## Supplementary Information

**Additional file 1: Supplementary information S1.**

**Additional file 2: Table S1.**

**Additional file 3: Table S2.**

**Additional file 4: Table S3.**

**Additional file 5.**

**Additional file 6: Table S4.**

## Data Availability

All single-cell RNA sequencing data are deposited in the Gene Expression Omnibus (GEO: GSE121455), and whole-genome sequencing data were deposited in European Genome-phenome Archive (EGA: EGAS00001003229). All these data are also deposited in the Genome Sequence Archive (GSA) with accession number PRJCA006394 [36].
