# Supplementary material for: Systematic evaluation of colorectal cancer organoid system by single-cell RNA-Seq analysis
Source: Genome Biol. 2022 Apr 28;23:106. doi: 10.1186/s13059-022-02673-3 (PMC9047329; doi:10.1186/s13059-022-02673-3)
Supplement: Supplementary file 1 — Additional file 1: Supplementary information S1. [file 13059_2022_2673_MOESM1_ESM.pdf]

Supplementary Information for

Systematic evaluation of colorectal cancer organoid system by single-cell RNA-Seq analysis

This file includes:

Additional file 1: Supplementary Figures

Fig. S1 Overview of sample collection and establishment of colorectal cancer organoid cultures.

Fig. S2 Tumor organoid cells and normal organoid cells that cultured in conditioned medium express different WNT signaling pathway target genes.

Fig. S3 Mitochondrial mutations of patients #2 and #3.

Fig. S4 Copy number variations of patient #1, #5 and #6 that were inferred by scRNA-seq data.

Fig. S5 Whole exome sequencing of organoids that cultured in chemical-defined and conditioned media.

Fig. S6 *In vitro* cultured organoids can maintain *in vivo* point mutations and CNVs of cancer cells.

Fig. S7 Cultured organoids tend to enrich for specific molecular features.

Fig. S8 Copy number variations of patient #4 and #7 that were inferred by scRNA-Seq data.

Additional file 2: Table. S1 Basic information of patients we collected, including pathological information, sequencing technology and culture information.

Additional file 3: Table. S2 UMAP clustering result of all single cells we analyzed.

Additional file 4: Table. S3 DEGs of *in vivo* normal and tumor epithelial cells and basic information of some DEGs and marker genes

Additional file 5: Table. S4 Summary table for genomic and epigenomic sequencing data, including WGS, WES and PBAT (DNA methylome) data

Fig S1

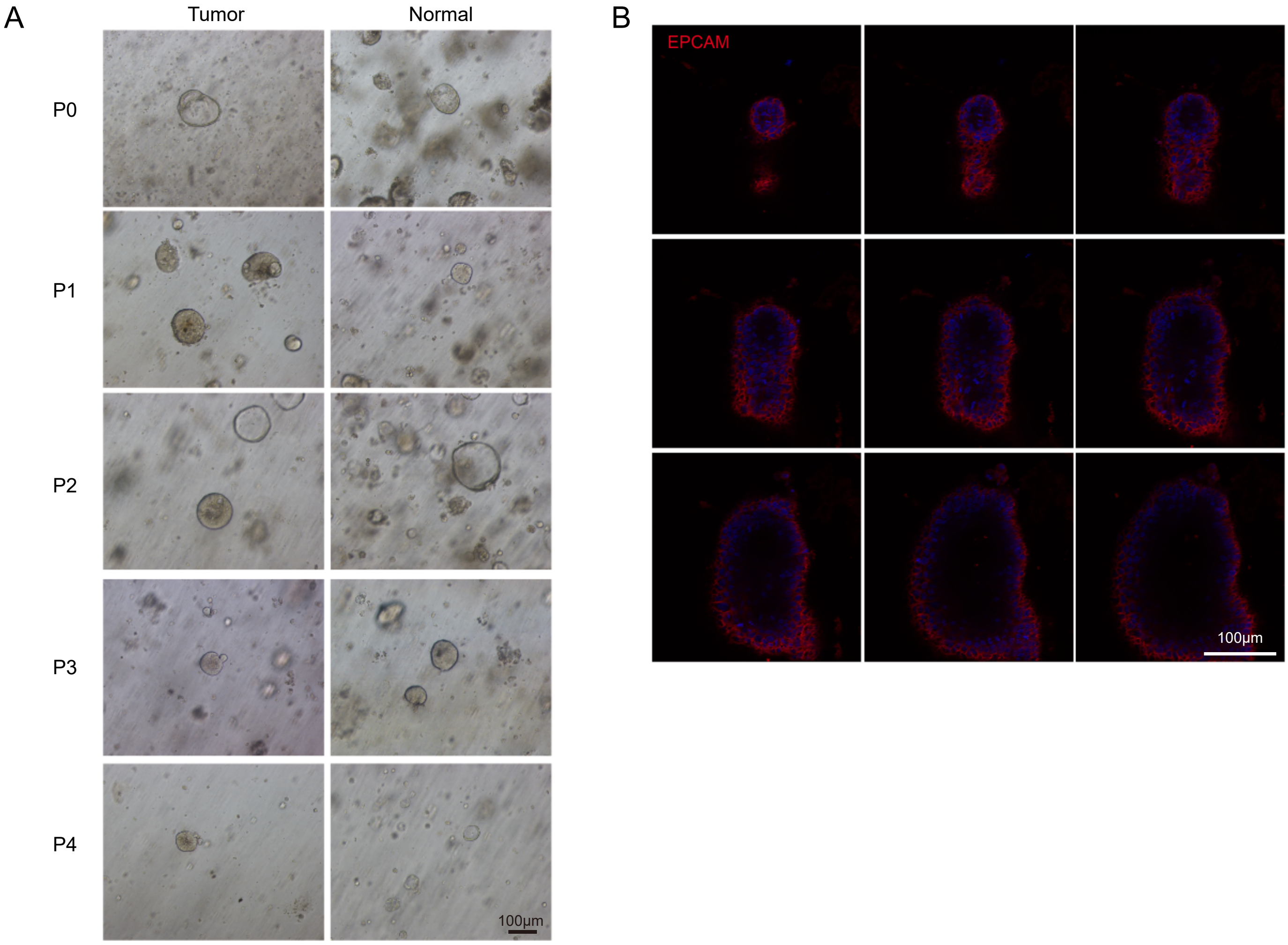

Fig. S1 Overview of sample collection and establishment of colorectal cancer organoid cultures.

(A) Representative bright-field images of tumor- and paired adjacent normal tissue-derived organoids of the patient from different passages. All scale bars, 100 µm. “P” stands for passage.

(B) Whole-mount immunofluorescent staining of EPCAM on normal tissue-derived organoids. Series of Z-axis optical sections are shown from left to right and from up to down. All scale bar, 100 µm.

Fig. S2

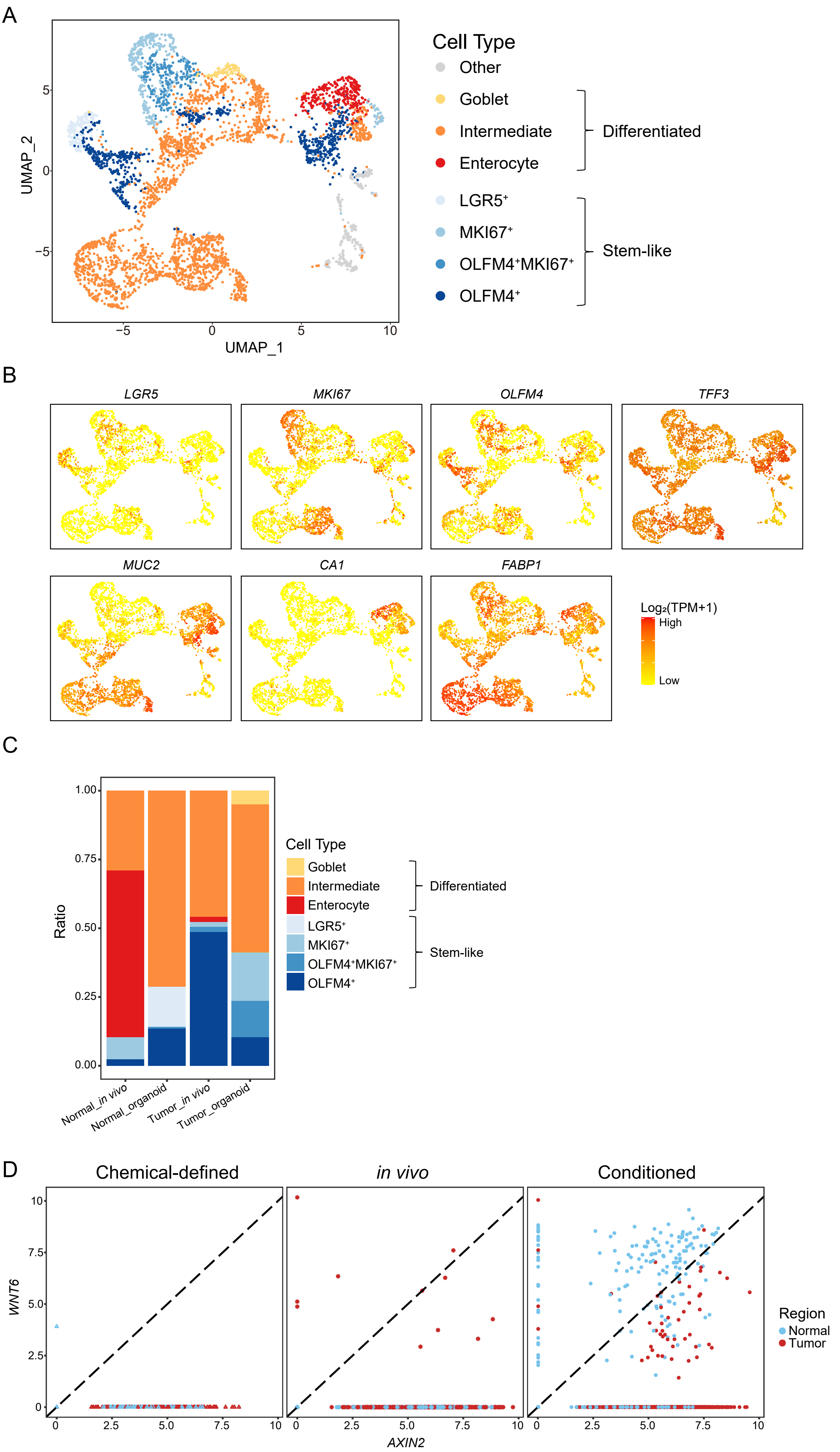

A
UMAP_2
UMAP_1
Cell Type
Other
Goblet
Intermediate
Enterocyte
Differentiated
LGR5⁺
MKI67⁺
OLFM4⁺MKI67⁺
OLFM4⁺
Stem-like
B
LGR5
MKI67
OLFM4
TFF3
MUC2
CA1
FABP1
Log₂(TPM+1)
High
Low
C
Ratio
Cell Type
Goblet
Intermediate
Enterocyte
Differentiated
LGR5⁺
MKI67⁺
OLFM4⁺MKI67⁺
OLFM4⁺
Stem-like
Normal_in vivo
Normal_organoid
Tumor_in vivo
Tumor_organoid
D
Chemical-defined
in vivo
Conditioned
WNT6
AXIN2
Region
Normal
Tumor

Fig. S2 Tumor organoid cells and normal organoid cells that were cultured in conditioned medium express different WNT signaling pathway target genes.

(A) UMAP clustering of cells and colors represent epithelial subtypes.

(B) Expression patterns of intestinal cell type markers were projected onto the UMAP plot. The colors from yellow to red represent expression levels from low to high. *OLFM4* and *LGR5*, intestinal stem/pluripotent cell markers; *MKI67*, cell proliferative marker; *CA1* and *FABP1*, enterocyte markers; *MUC2* and *TFF3*, goblet cell markers. Basic information of these cell type markers can be found in Supplementary Table S3.

(C) Bar plot showing the distribution of epithelial subtypes in different conditions.

(D) Scatterplot showing the expression patterns of the WNT signaling pathway target genes *WNT6* and *ANXIN2* in each individual cell. Different colors represent cells collected from normal or tumor regions.

Fig. S3

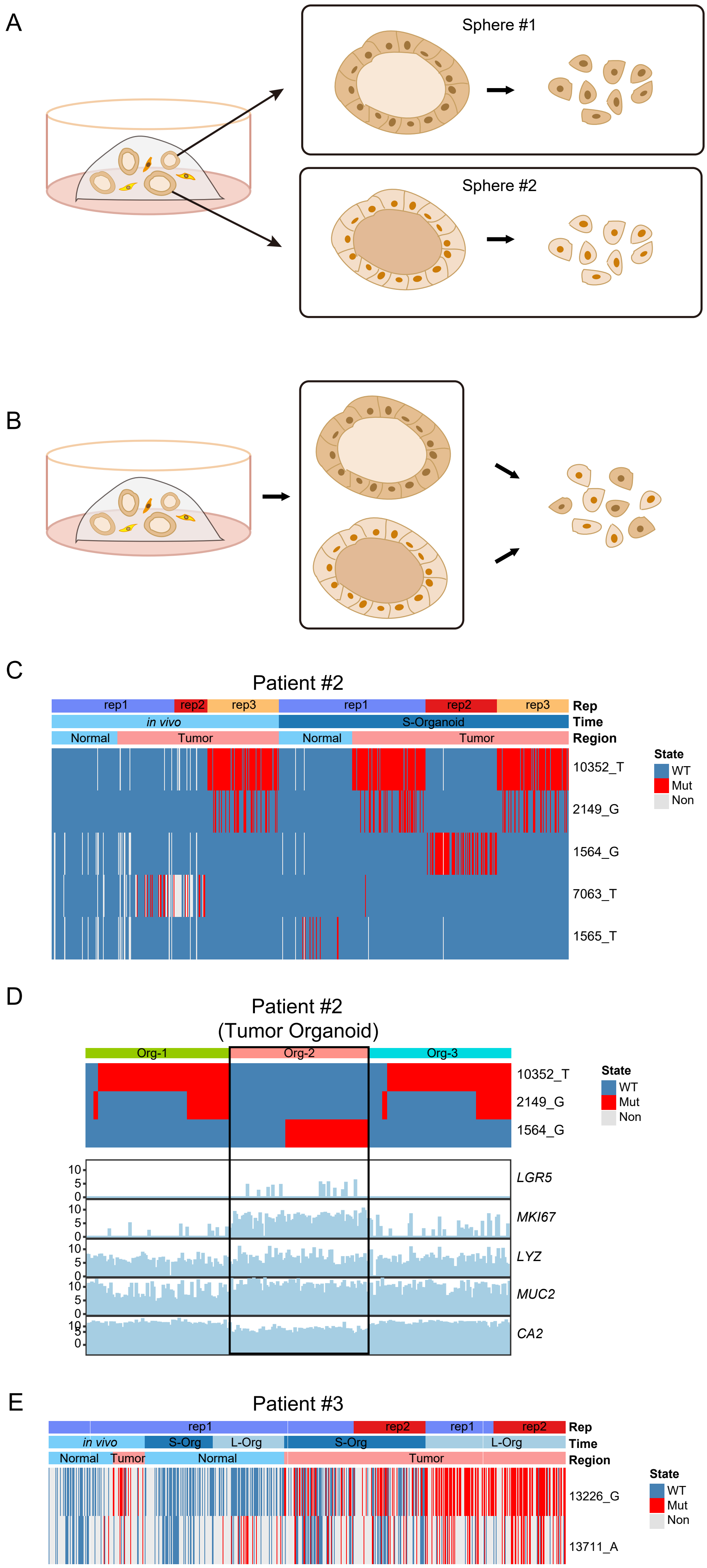

A
Sphere #1
Sphere #2
B
C
Patient #2
rep1
rep2
rep3
rep1
rep2
rep3
Rep
in vivo
S-Organoid
Time
Normal
Tumor
Normal
Tumor
Region
10352_T
2149_G
1564_G
7063_T
1565_T
State
WT
Mut
Non
D
Patient #2
(Tumor Organoid)
Org-1
Org-2
Org-3
10352_T
2149_G
1564_G
State
WT
Mut
Non
LGR5
MKI67
LYZ
MUC2
CA2
E
Patient #3
rep1
rep2
rep1
rep2
Rep
in vivo
S-Org
L-Org
S-Org
L-Org
Time
Normal
Tumor
Normal
Tumor
Region
13226_G
13711_A
State
WT
Mut
Non

Fig. S3 Mitochondrial mutations of patients #2 and #3.

(A) Schematic diagram showing single cell picking strategies of patient #1 and #2 for *in vitro* cultured organoids.

(B) Schematic diagram showing single cell picking strategies of other patients for *in vitro* cultured organoids.

(C) Heatmap showing mitochondrial mutation of patient #2 (P2). Red represents mutant site, blue represents wild type and grey represents site with reads lower than 9. Chem, chemical-defined medium; Cond, conditioned medium.

(D) Heatmap showing the mitochondrial mutations in tumor organoid cells of patient #2 that cultured in chemical-defined medium. Cells of three single organoid spheres were showed. Bar plot showing the corresponding expression levels of intestinal cell type markers. *OLFM4*, pluripotent cell marker; *MKI67*, cell proliferation marker; *LYZ*, Paneth cell marker; *MUC2*, goblet cell marker; *CA2*, enterocyte marker

(E) Heatmap showing mitochondrial mutation of patient #3. Red represents mutant site, blue represents wild type and grey represents site with reads lower than 9. Chem, chemical-defined medium; Cond, conditioned medium.

Fig. S4

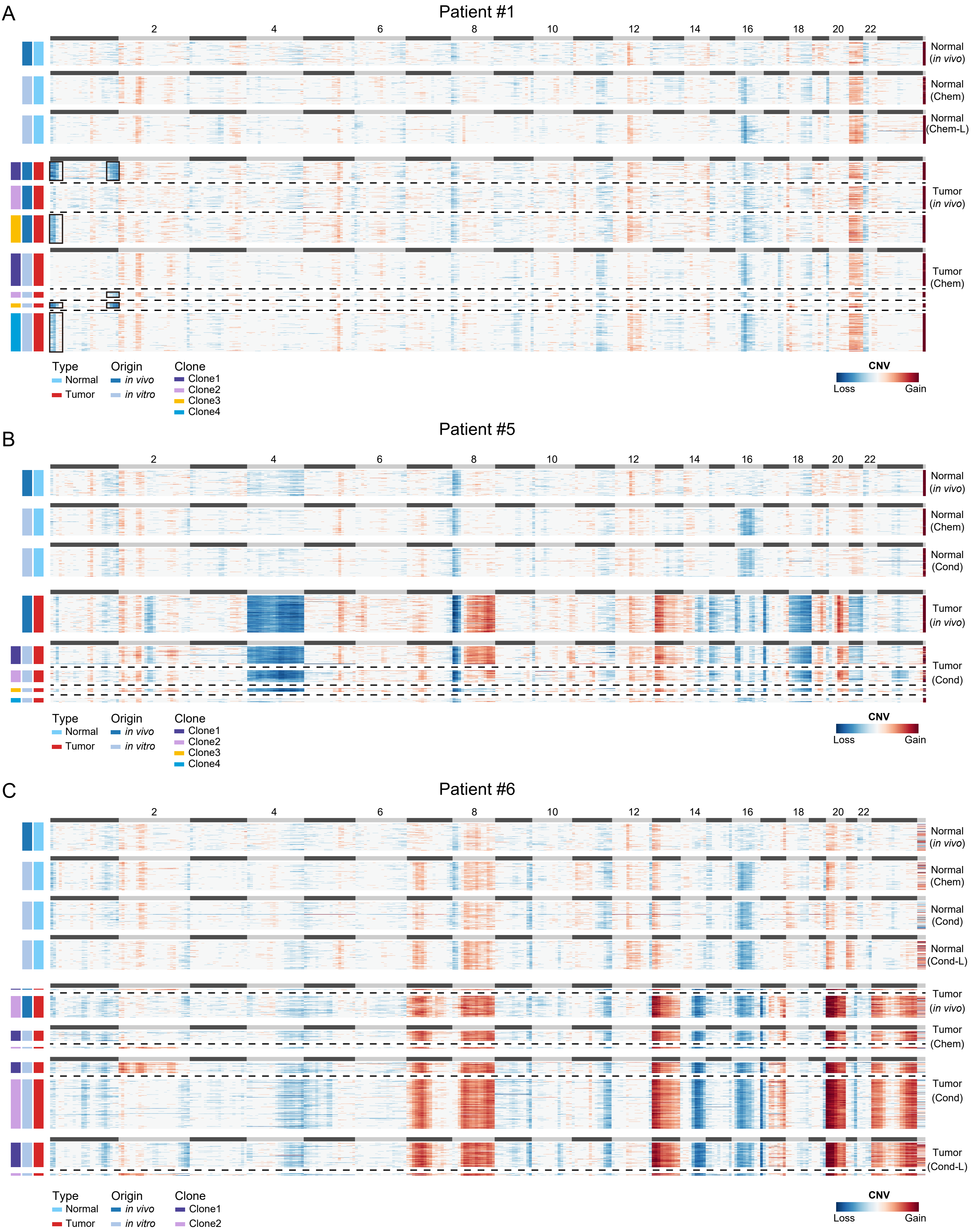

A
Patient #1
2 4 6 8 10 12 14 16 18 20 22
Normal (in vivo)
Normal (Chem)
Normal (Chem-L)
Tumor (in vivo)
Tumor (Chem)
Type
Normal
Tumor
Origin
in vivo
in vitro
Clone
Clone1
Clone2
Clone3
Clone4
CNV
Loss
Gain
B
Patient #5
2 4 6 8 10 12 14 16 18 20 22
Normal (in vivo)
Normal (Chem)
Normal (Cond)
Tumor (in vivo)
Tumor (Cond)
Type
Normal
Tumor
Origin
in vivo
in vitro
Clone
Clone1
Clone2
Clone3
Clone4
CNV
Loss
Gain
C
Patient #6
2 4 6 8 10 12 14 16 18 20 22
Normal (in vivo)
Normal (Chem)
Normal (Cond)
Normal (Cond-L)
Tumor (in vivo)
Tumor (Chem)
Tumor (Cond)
Tumor (Cond-L)
Type
Normal
Tumor
Origin
in vivo
in vitro
Clone
Clone1
Clone2
CNV
Loss
Gain

Fig. S4 Copy number variations of patient #1, #5 and #6 that were inferred by scRNA-Seq data.

(A) CNV patterns of *in vivo* and *in vitro* cultured tumor cells of patient #1 (P1). Blue, deletion. White, normal. Red, amplification. There were three and four subclones were identified for tumor cells *in vivo* and *in vitro* respectively. We clustered the cells based on their CNV patterns, and we defined cells possessing different CNVs as distinct clones.

(B) CNV patterns of *in vivo* and *in vitro* cultured tumor cells of patient #5 (P5). Blue, deletion. White, normal. Red, amplification. There were four subclones were identified for tumor cells *in vitro*.

(C) CNV patterns of *in vivo* and *in vitro* cultured tumor cells of patient #6 (P6). Blue, deletion. White, normal. Red, amplification. Two subclones were identified for both *in vivo* and *in vitro* tumor cells.

Fig. S5

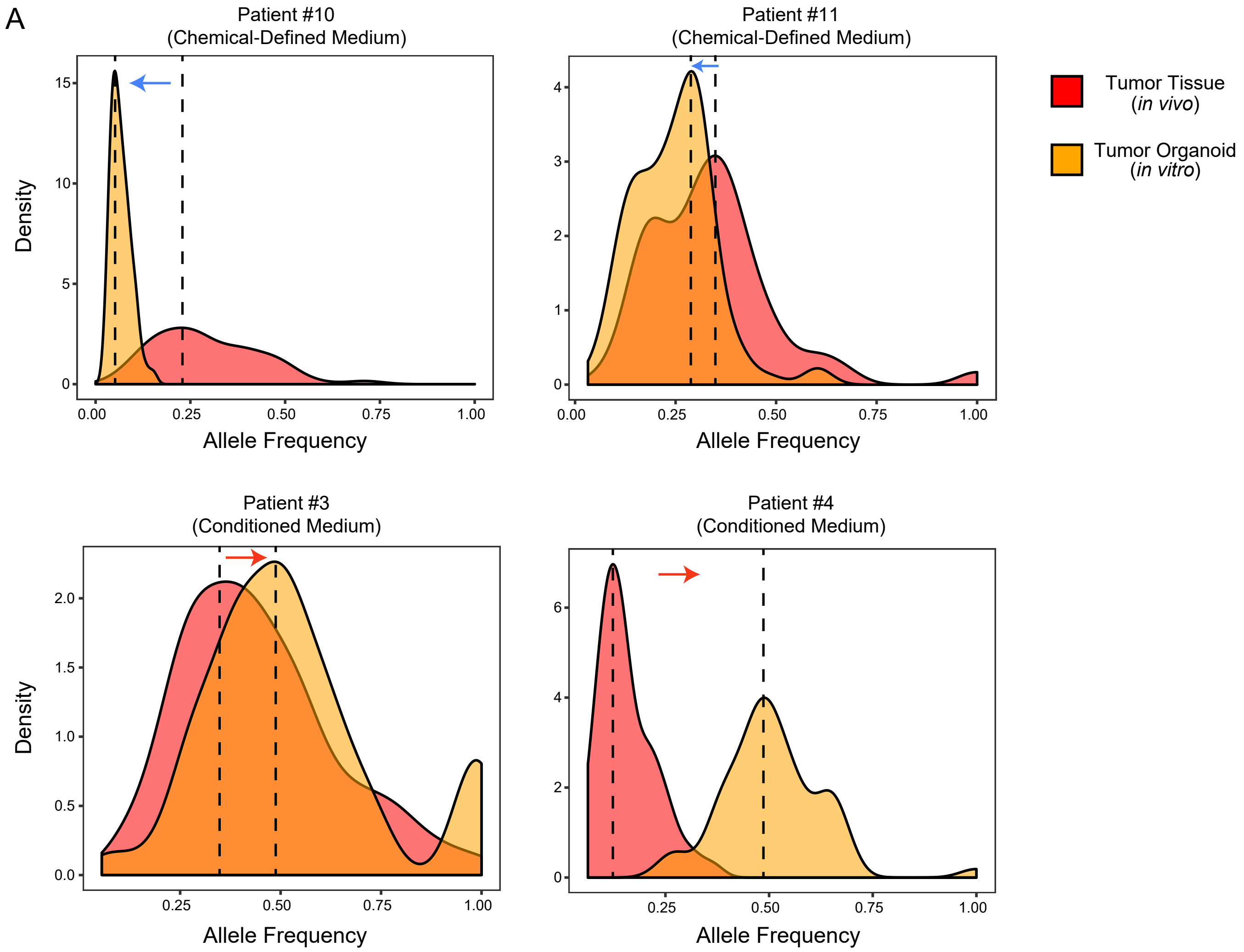

A
Patient #10
(Chemical-Defined Medium)
Patient #11
(Chemical-Defined Medium)
Patient #3
(Conditioned Medium)
Patient #4
(Conditioned Medium)
Density
Allele Frequency
0.00
0.25
0.50
0.75
1.00
15
10
5
0
4
3
2
1
2.0
1.5
1.0
0.5
0.0
6
Tumor Tissue
(in vivo)
Tumor Organoid
(in vitro)

Fig. S5 Whole exome sequencing of organoids that were cultured in chemical-defined and conditioned media.

(A) Density plot showing the allele frequency of somatic mutations that shared by in vivo and in vitro tumor cells. Tumor organoids of patient #10 and #11 (P10 and P10) were cultured in chemical-defined medium, while tumor organoids of patient #3 and #4 were cultured in conditioned medium.

Fig. S6

A

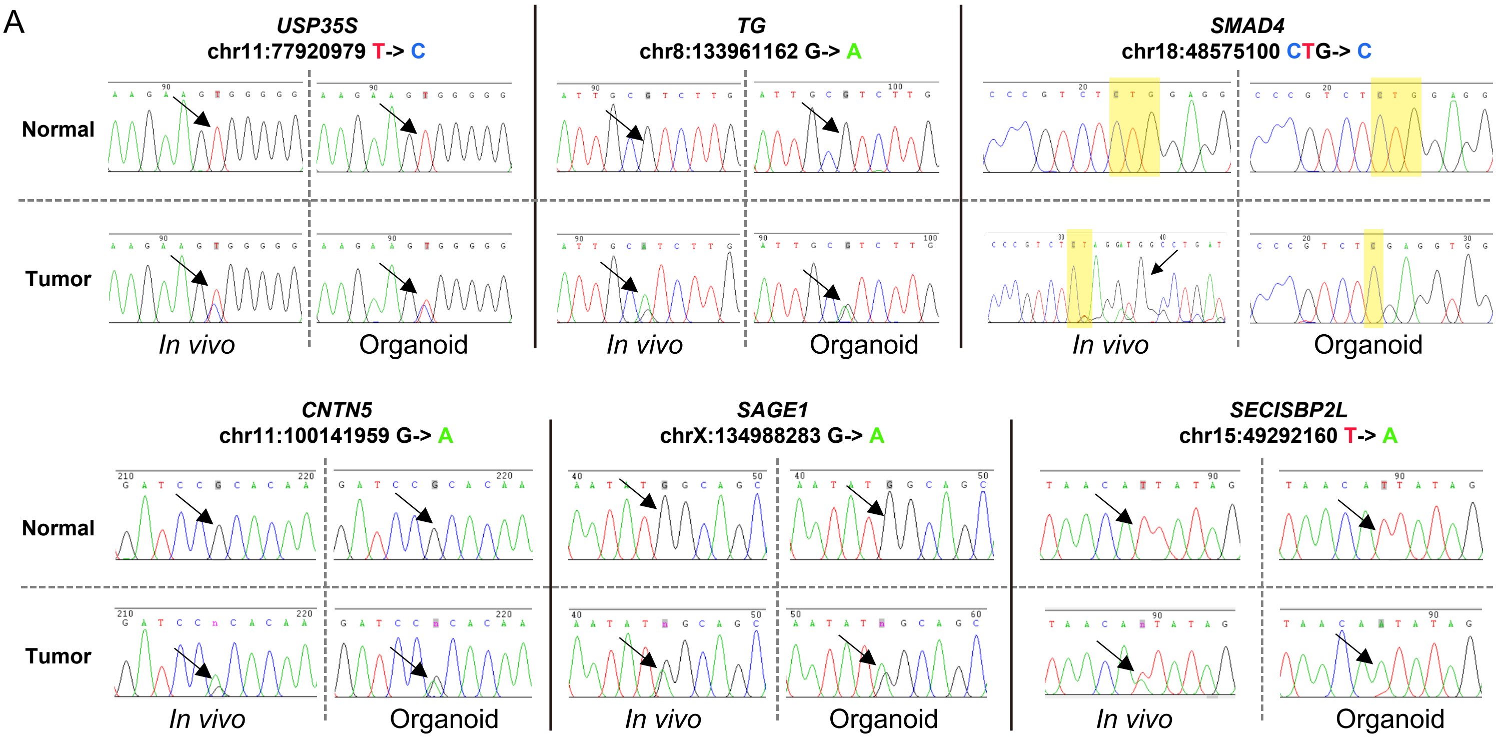
USP35S
chr11:77920979 T-> C
TG
chr8:133961162 G-> A
SMAD4
chr18:48575100 CTG-> C
Normal
Tumor
In vivo
Organoid
CNTN5
chr11:100141959 G-> A
SAGE1
chrX:134988283 G-> A
SECISBP2L
chr15:49292160 T-> A

B

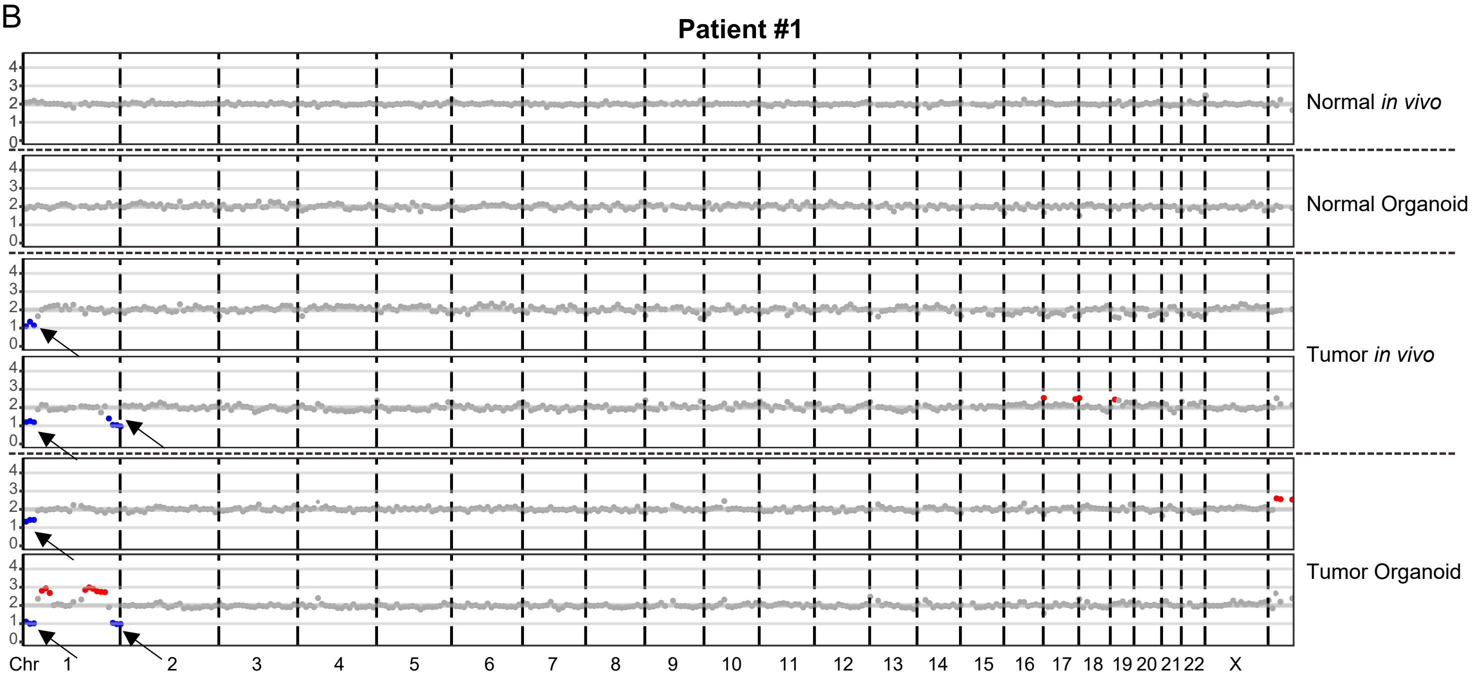
Patient #1
Normal in vivo
Normal Organoid
Tumor in vivo
Tumor Organoid
Chr 1 2 3 4 5 6 7 8 9 10 11 12 13 14 15 16 17 18 19 20 21 22 X

Fig. S6 *In vitro* cultured organoids can maintain *in vivo* point mutations and CNVs of cancer cells.

(A) Point mutation verification of six mutation sites for patient #1 *in vivo* tissues and derived organoids by Sanger sequencing. Arrows indicate the sites of mutation.

(B) Bulk whole genome sequencing (WGS) of *in vivo* and *in vitro* tumor cells and normal cells for patient #1. Blue, deletion. Red, amplification. Arrows indicate the CNV site.

Fig S7

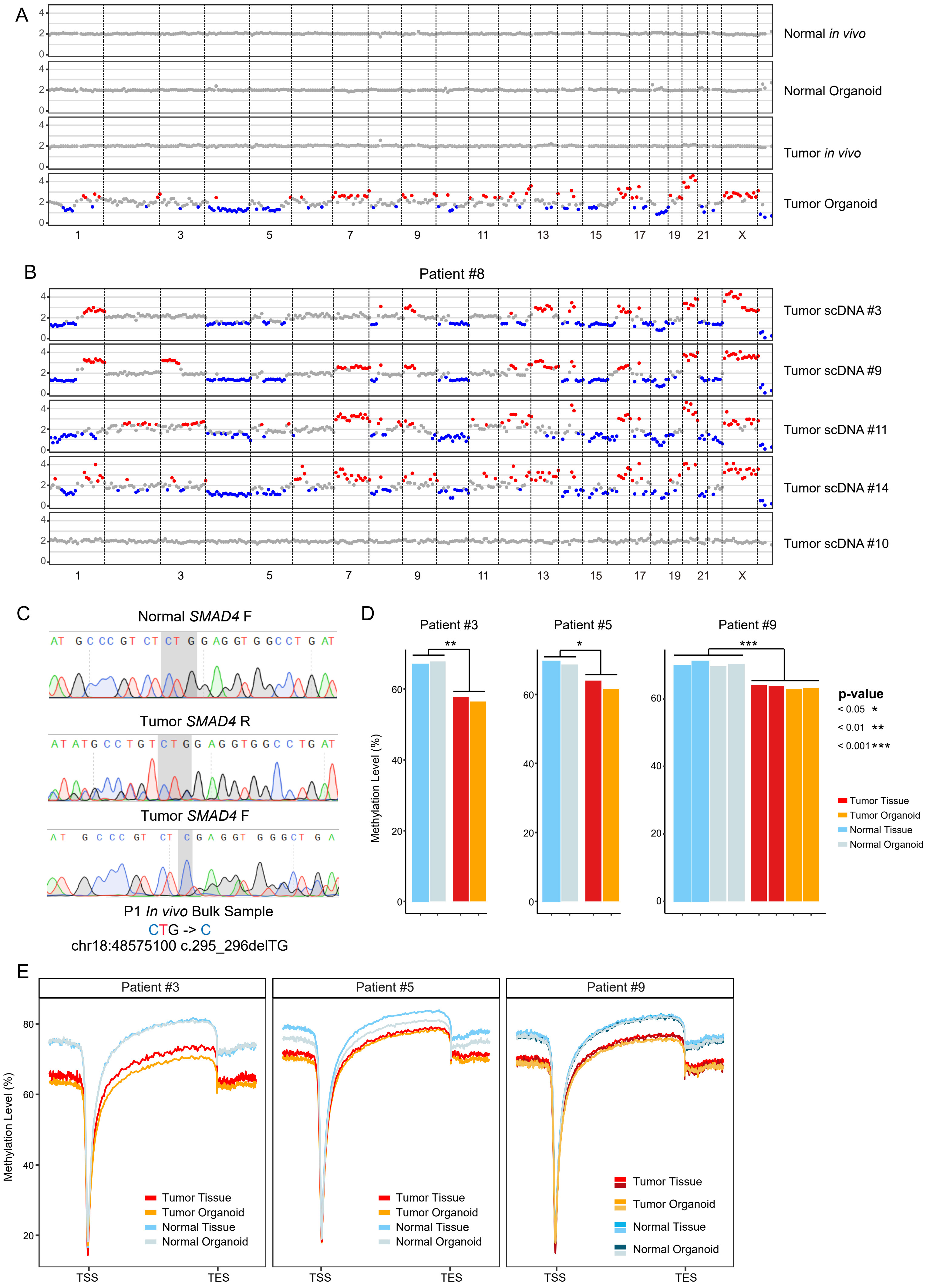
A
Patient #8
Normal in vivo
Normal Organoid
Tumor in vivo
Tumor Organoid
1
3
5
7
9
11
13
15
17
19
21
X
B
Patient #8
Tumor scDNA #3
Tumor scDNA #9
Tumor scDNA #11
Tumor scDNA #14
Tumor scDNA #10
C
Normal SMAD4 F
Tumor SMAD4 R
Tumor SMAD4 F
P1 In vivo Bulk Sample
CTG -> C
chr18:48575100 c.295_296delTG
D
Patient #3
Patient #5
Patient #9
Methylation Level (%)
p-value
< 0.05 *
< 0.01 **
< 0.001 ***
Tumor Tissue
Tumor Organoid
Normal Tissue
Normal Organoid
E
Patient #3
Patient #5
Patient #9
Methylation Level (%)
TSS
TES
Tumor Tissue
Tumor Organoid
Normal Tissue
Normal Organoid

Fig. S7 Cultured organoids tend to enrich for specific molecular features.

(A) CNVs of patient #8 at bulk levels for magnetic-activated cell sorting (MACS)-enriched EPCAM$^+$ tumor epithelial cells and adjacent normal cells as well as derived organoid cultures.

(B) CNVs of patient #8 at the single-cell level for tumor epithelial cells *in vivo*. In total, we sequenced 20 cells and other cells we not showed exhibited similar pattern as cell #10.

(C) Point mutation verification of *SMAD4* for patient #1 *in vivo* tissues and derived organoids by Sanger sequencing.

(D) Bar plot showing the global DNA methylation ratio of *in vivo* and *in vitro* cultured tumor cells and normal cells. All organoids of these patients were constructed in conditioned medium.

(E) Line plot showing the DNA methylation ration around transcriptional start site (TSS), gene body and transcriptional end site (TES). The x axis represents the relative position of genes and y axis represents the average DNA methylation levels. Different color represents cells with different origins. It reflects that the organoids cultured *in vitro* maintained the global DNA methylation signatures of corresponding cells *in vivo*. *In vivo* normal tissues and in vitro normal tissue-derived organoids have similar DNA methylation profiles, and the methylation levels are higher than both *in vivo* tumor cells and *in vitro* tumor-derived organoids as expected.

Fig. S8

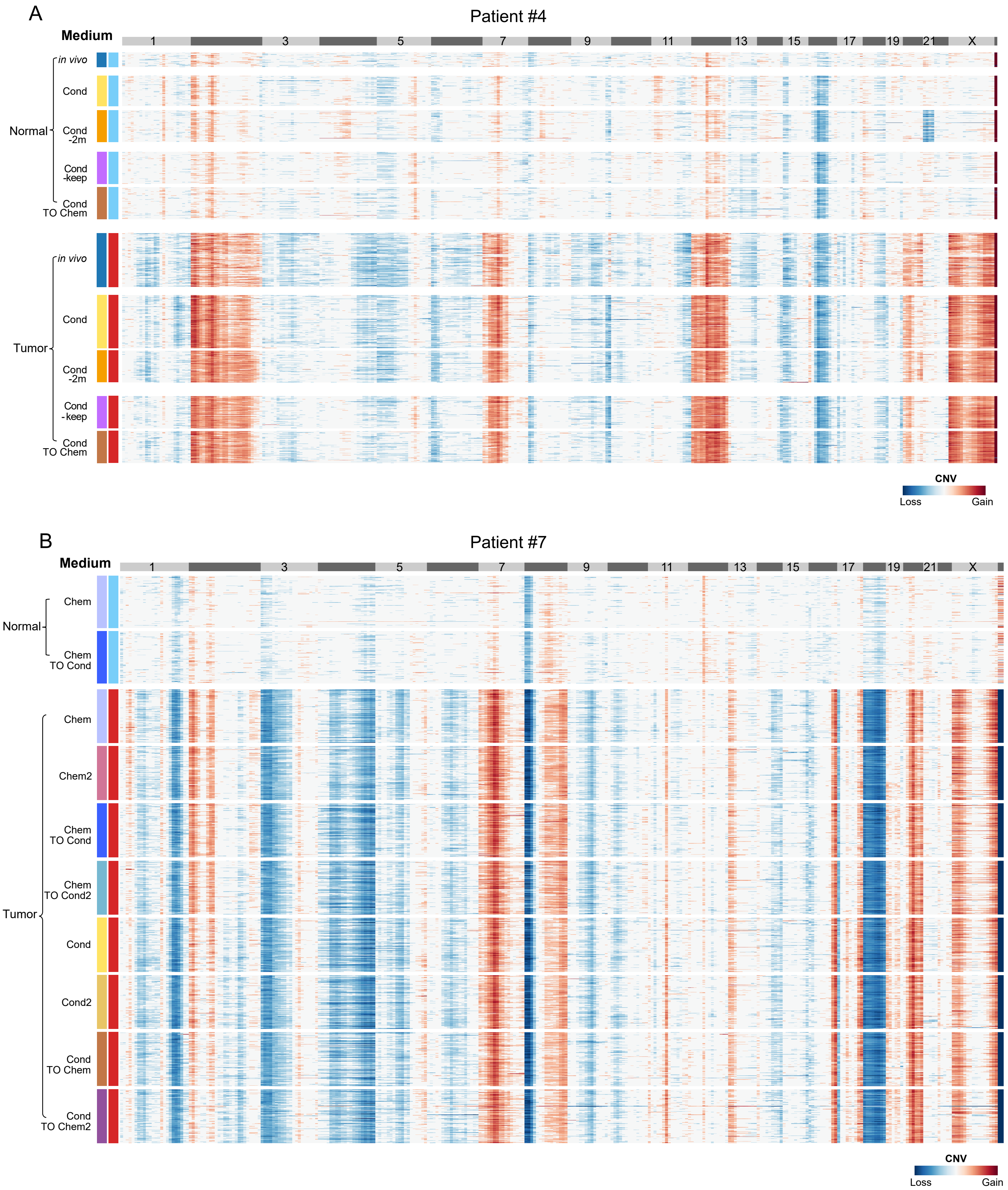
A
Patient #4
Medium
1
3
5
7
9
11
13
15
17
19
21
X
Normal
in vivo
Cond
Cond
-2m
Cond
-keep
Cond
TO Chem
Tumor
in vivo
Cond
Cond
-2m
Cond
- keep
Cond
TO Chem
CNV
Loss
Gain
B
Patient #7
Medium
1
3
5
7
9
11
13
15
17
19
21
X
Normal
Chem
Chem
TO Cond
Tumor
Chem
Chem2
Chem
TO Cond
Chem
TO Cond2
Cond
Cond2
Cond
TO Chem
Cond
TO Chem2
CNV
Loss
Gain

Fig. S8 Copy number variations of patient #4 and #7 that were inferred by scRNA-Seq data.

(A) CNV patterns of *in vivo* and *in vitro* cultured tumor cells and normal cells of patient #4. Blue, deletion. White, normal. Red, amplification.

(B) CNV patterns of *in vivo* and *in vitro* cultured tumor cells and normal cells of patient #7. Blue, deletion. White, normal. Red, amplification.
