# Supplementary material for: Systematic evaluation of colorectal cancer organoid system by single-cell RNA-Seq analysis
Source: Genome Biol. 2022 Apr 28;23:106. doi: 10.1186/s13059-022-02673-3 (PMC9047329; doi:10.1186/s13059-022-02673-3)
Supplement: Supplementary file 5 — Additional file 5. [file 13059_2022_2673_MOESM5_ESM.docx]

Review History

**First round of review**

**Reviewer 1**

**Were you able to assess all statistics in the manuscript, including the appropriateness of statistical tests used?**

**Yes**

**Comments to author:**

The paper used high-resolution integrated multiomics of single-cell sequencing, and analyzed the features of the organoids derived from colorectal cancer with 2 culture systems. It studied the message on the similarities and differences between the 2 systems (the conventional defined chemical system, and the conditioned system), the different tissues (cancer tissues and their nearby “normal” tissues), at different culture time snapshots, for their transcriptomic, and genomic profiles (CNA pattern, SNA), and epigenomics (DNA methylation at genome wide), in which the DNA methylation is also regarded as a genomic feature, at single cell resolution or / and bulk cell level.

The paper presented a set of interesting results/conclusions. 1) the organoids derived from the 2 types of resources overall recapitulated the transcriptomic pattern, exampled by several gene signatures of the cancer. 2) the organoids derived from normal tissues exhibited some tumor-like features on the whole transcriptome level, but retained normal genomes. In the discussion it summarized in more detail: However, the genomic features and epigenomic features, such as CNVs, point mutations and DNA methylation patterns, of normal tissue-derived organoids still remain normal. [Concerns #1: this conclusion may have a little problem]. 3).the conditioned medium outperformed the chemical defined medium in long-term culture of tumor epithelial cells (in the Abstract). And in the discussion, it further described as that the chemical defined medium is more conducive to the proliferation of normal epithelial cells in vitro, while tumor cells and normal epithelial cells have comparable proliferation rates in conditioned medium, and that conditioned medium can better reflect the difference of the degree of differentiation between tumor cells and normal epithelial cells in vivo than chemical defined medium. 4) when the 2 culture mediums for the organoids were mutually exchanged, the organoid cells maintained the main transcriptome characteristics of the original medium. And it further concluded that cultured medium that was initially used to derive the organoids plays a very critical role in the gene expression profiles of the organoids.

Major Concerns:

1, regarding the result/conclusion#2 mentioned above. Firstly, it is well understanding that if transcriptomic profile changes, it must be regulated by epigenomic or/and genomic elements, esp. these epigenomic regulatory elements, such as DNA methylation, chromatin conformation (open chromatin, histone modification, …), miRNA, etc. Secondly the genomic/epigenomic analysis for these samples were rather rough and in general (FigS7D, E). Therefore, it is reasonable to argue that some subtle and definite changes on epigenomic aspect should be involved, which may become visible with a deeper analysis on the DNA methylation data that is available, which analysis is some insufficient at present, ex. genomic profile and epigenomic profile may be investigated and considered separately; although in a broad sense, epigenomics or DNA methylation can be classified under genomics, and even transcriptomics is also a part of genomics. The result seems true that the “DNA methylation level” overall keeps unchanged. Nevertheless, what do the “DNA methylation pattern” (line#222) refer (when it is claimed that cultured organoids can preserve the genomic features of the tissues in vivo, including …… DNA methylation patterns).? How about the associated regulatory DNA elements of the genes changed between the in vivo and the organoid of the normal tissues?

2, In quite a few cases, the result description, and the demonatration/argument for the conclusion, reflecting the analysis, is very rough, not in sufficient detail, for example (but not limited to these examples):

1. line#68-69, “several epithelial cell markers”, what are these markers? Why these markers?
2. Line#86, annotated as “epithelial cells, immune cells and mesenchymal cells”, this classification seems too general. With more detailed clustering, some difference between conditions may become visible, especially the epithelial cells.
3. Line#86, furthermore, on the Right side of the FigS2B, it shows different pattern (sub set of cells) between the In vivo tumor and the organoid tumor. How do the author interpret it?
4. .line #100, which genes are important? Why significant and make sense in biology?
5. Line#189-190, the result Fig 4F as well as the conclusion (therefore…) are not well interpreted or demonstrated.
6. Line#199, How is it verified? Where is the data?
7. Line#244, “due to lacking cell-cell interaction”, is it true?
8. Line#563-564, Fig1(E): none of these markers is described for the function or significance.
9. Line#579, Fig2(E): Is there any biological significance for these genes listed? Particularly the red genes on the right side?
10. Line#672, FigS4: which and where are the 3 clones, or 4 clones?
11. Line#703, FigS7E: What do these data tell? Why these data are shown??
12. The paragraph started at line#216, with the DNA methylation, only a general profile is given, which is truly informative. However, is there any particular change for the normal tissue between the in vivo biopsy and the organoid? how about the answer of the tumor biopsy?
13. In the Abstract, regarding genomic (including eigenomic) profiling, the description does not give any detailed message, such as CNV or SNV mutation, or DNA methylation. This way a reader would not know better what you refer to

Minor Concerns:

1, About the technology

1)

For single cell CNV calling, 2 methods are used for the set of same set of samples studied, scRNA-seq-inferred CNV, and MALBAC analyzed CNV. What are the correlation, consistence or inconsistence between these 2 methods?

1. For scRNA-seq, STRT method is used, so not very many cells are obtained with single cell data. What are the key advantages of STRT that the authors took so as to use STRT instead of 10x genomics or BD Rhapsody in this project?

2, About spelling

1. Line#243, “… cell line which can not …” may be better to be” cell line, which can’t ” (with a comma “,” before which”)
2. Line#610, Is it correct “bottom left”?
3. Fig4H, “enrich for epithelial cell”, or “enrich for epithelial cells” (cell with “s” or without “s”)

Review Conclusion:

The topic discussed in this manuscript is important. Overall this manuscript gives a set of interesting results and conclusions, which are novel, and very valuable to the researchers in the field.

The experiments basically are reliable, and the conclusions are overall convincing. However, for some presented results and conclusions, the analysis/ demonstration is relatively simple, crude and insufficient. Therefore more deep analysis (of the data), detailed description (of the results), demonstration and interpretation are necessary. This further effort should make the conclusions more accurate and solid.

Suggestion for decision: Minor Revision

**Reviewer 2**

**Were you able to assess all statistics in the manuscript, including the appropriateness of statistical tests used?**

Yes

**Comments to author:**

In this manuscript, Wang and colleagues report a comparative genomic analysis of tumor and adjacent normal tissue-derived 3D organoids from patients with colorectal cancer. Using scRNA-seq, bulk and single-cell whole genome sequencing, whole exome sequencing (WES), whole genome bisulfite sequencing and Sanger sequencing, organoids were compared with each other, with their in vivo counterparts, and across distinct culture conditions. In doing so, the authors identified negligible genomic differences between tumor-derived organoids and paired in vivo tumor samples. In contrast, adjacent normal tissue-derived organoids exhibited some transcriptional hallmarks of primary tumor samples regardless of culture medium, yet largely maintained normal genomes. Additionally, the authors found that (i) a conditioned culture medium was superior for long-term culture of tumor epithelial cells and that (ii) characteristics observed in the original culture medium were generally maintained after exchanging one culture medium for another.

Overall, this paper, while lacking in innovation, includes an important message for the field: tumor-derived organoids, but not adjacent normal tissue-derived organoids, tend to faithfully recapitulate in vivo genomic characteristics. Optimizing culture conditions to better preserve in vivo features of normal adjacent tissue-derived 3D organoids represents a critical unmet need.

Despite the strengths of this study, there are several critical shortcomings that should be addressed.

Major comments:
1. The authors' analysis of cell composition by scRNA-seq is rather pedestrian, with only three major lineages identified (epithelial cells, immune cells and mesenchymal cells). I suggest the use of granular marker genes, unsupervised clustering, and/or reference guided annotation to delineate cellular composition at considerably higher resolution in order to precisely distinguish cellular heterogeneity within each of these three compartments. For example, both tumor and normal colon epithelial cells, as well as stromal and immune subsets, have been extensively characterized by previous scRNA-seq studies (e.g., PMID 32451460), with marker genes and reference profiles that could be applied here. Such data could also serve as an anchor for Seurat integration.
2. Critical methodological details for scRNA-seq analysis, WGS analysis, WES analysis, and methylation analysis are missing. This is rather surprising given the focus of the paper. In general, references to previous literature ("as previously described") do not facilitate reproducibility. All methods, parameters used, software, and software version numbers should be provided in a self-contained manner. See related comments below.
3. The network diagram in Figure 3A is cryptic. What tumor-specific regulatory network is shown? How was the regulatory network identified? Which genes are shown? Highlighting at least several key genes and providing a legend for the color scale would be helpful.
4. I suggest that the authors perform gene set enrichment and/or another pathway analysis method to more systematically explore transcriptional differences between organoid sources, organoid vs. in vivo tissues, and culture conditions.
5. Details of DNA sequencing data are missing, for example the number of reads obtained per sample, the number/fraction properly paired and on-target, uniformity statistics for bulk/single-cell WGS, etc.
6. The scRNA-seq preprocessing steps are unclear. Did the authors use % reads mapped to mitochondrial genes as a filtration criterion, as commonly applied to eliminate dead/dying cells? It appears not. The step involving the use of "cell-to-cell correlation (top 2 correlation higher than 0.6)" is odd; I assume this was done to remove doublets? No justification is provided for the approach or the threshold, and I wonder why the authors did not instead use an established doublet detection method, such as Scrublet.
7. Line 412: "Low-quality and index contaminate reads were first removed from the raw paired-end sequence data." What constitutes low quality?
8. Line 415: '10 M windows'. Windows of equal size?
9. Lines 416-417: Generally, copy number inference is performed against a paired germline control. By their methodological description, it is unclear whether the authors employed germline controls. The rationale for the read normalization approach is unclear and should be both justified and elaborated.
10. Lines 419-426: The entire WES pipeline should be specified in detail.
11. How were mitochondrial mutations identified? What quality control steps were employed?
12. How was methylation calling performed?
13. Line 310: "Organoid culture was performed as previously described". This is missing a reference and should ideally include a full description of the procedure here.

Minor comments:
1. UMAP in Figure 1D: This is a result of integration across samples, yet sample ID and culture conditions are relegated to Fig S1A. For coherence, I would suggest adding Fig S1A to Figure 1.
2. Figure 5 heat map: expression level values are not aligned with the color bar in the legend.

**Authors Response**

**Point-by-point responses to the reviewers’ comments:**

**Reviewer 1**

The paper used high-resolution integrated multiomics of single-cell sequencing, and analyzed the features of the organoids derived from colorectal cancer with 2 culture systems. It studied the message on the similarities and differences between the 2 systems (the conventional defined chemical system, and the conditioned system), the different tissues (cancer tissues and their nearby “normal” tissues), at different culture time snapshots, for their transcriptomic, and genomic profiles (CNA pattern, SNA), and epigenomics (DNA methylation at genome wide), in which the DNA methylation is also regarded as a genomic feature, at single cell resolution or / and bulk cell level. The paper presented a set of interesting results/conclusions. 1) the organoids derived from the 2 types of resources overall recapitulated the transcriptomic pattern, exampled by several gene signatures of the cancer. 2) the organoids derived from normal tissues exhibited some tumor-like features on the whole transcriptome level, but retained normal genomes. In the discussion it summarized in more detail: However, the genomic features and epigenomic features, such as CNVs, point mutations and DNA methylation patterns, of normal tissue-derived organoids still remain normal. [Concerns #1: this conclusion may have a little problem]. 3).the conditioned medium outperformed the chemical defined medium in long-term culture of tumor epithelial cells (in the Abstract). And in the discussion, it further described as that the chemical defined medium is more conducive to the proliferation of normal epithelial cells in vitro, while tumor cells and normal epithelial cells have comparable proliferation rates in conditioned medium, and that conditioned medium can better reflect the difference of the degree of differentiation between tumor cells and normal epithelial cells in vivo than chemical defined medium. 4) when the 2 culture mediums for the organoids were mutually exchanged, the organoid cells maintained the main transcriptome characteristics of the original medium. And it further concluded that cultured medium that was initially used to derive the organoids plays a very critical role in the gene expression profiles of the organoids.

*Response: We sincerely thank the reviewer for the positive comments on our manuscript and also for the very constructive suggestions on how to improve the manuscript. Following the reviewer’s suggestions, we have revised our manuscript accordingly.*

Major concerns:

1, regarding the result/conclusion#2 mentioned above. Firstly, it is well understanding that if transcriptomic profile changes, it must be regulated by epigenomic or/and genomic elements, esp. these epigenomic regulatory elements, such as DNA methylation, chromatin conformation (open chromatin, histone modification, …), miRNA, etc. Secondly the genomic/epigenomic analysis for these samples were rather rough and in general (FigS7D, E). Therefore, it is reasonable to argue that some subtle and definite changes on epigenomic aspect should be involved, which may become visible with a deeper analysis on the DNA methylation data that is available, which analysis is some insufficient at present, ex. genomic profile and epigenomic profile may be investigated and considered separately; although in a broad sense, epigenomics or DNA methylation can be classified under genomics, and even transcriptomics is also a part of genomics. The result seems true that the “DNA methylation level” overall keeps unchanged. Nevertheless, what do the “DNA methylation pattern” (line#222) refer (when it is claimed that cultured organoids can preserve the genomic features of the tissues in vivo, including …… DNA methylation patterns).? How about the associated regulatory DNA elements of the genes changed between the in vivo and the organoid of the normal tissues?

*Response: We thank the reviewer for the insightful comments, and we agreed with the reviewer that the conclusions on the DNA methylation section are not rigorous. As the reviewer mentioned, even if the global DNA methylation levels are similar, there may be subtle differences in methylation at some genes or regulatory elements. Following the reviewer suggestion, we calculated the methylation levels of different regulatory elements, it showed that except CGI, normal tissues and normal tissue-derived organoids have higher methylations in other regulatory elements compared with tumor and tumor-derived organoids (Figure R1). However, since our sequencing depth is relatively shallow (if sequencing the samples to high depth (for example, 30X), it will be very expensive), and there are no or just two replicates for each condition, which is not enough for performing statistically test, we have toned down the conclusions of this part, and we are very grateful to the reviewers for their constructive suggestions.*


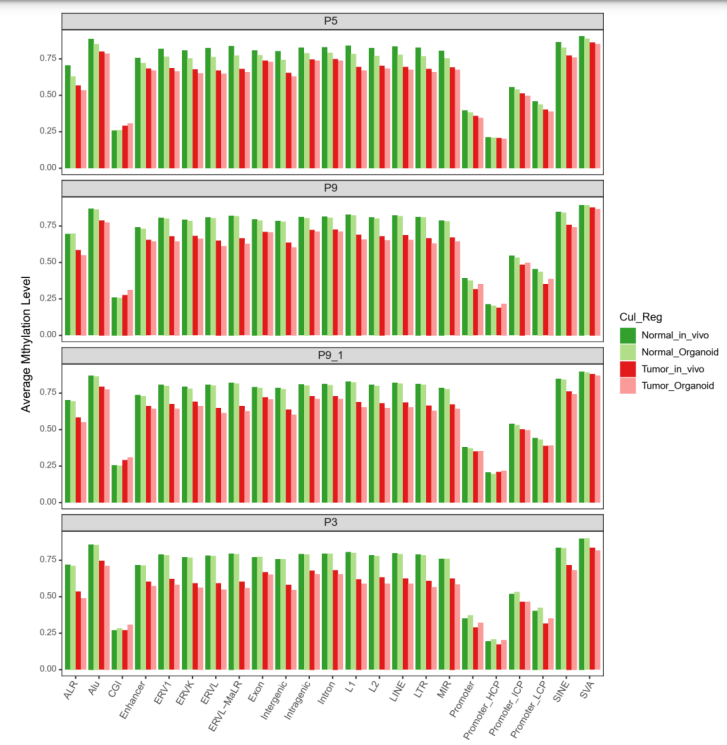


*Figure R1 Bar plot showing the average methylation levels of different regulatory elements.*

2, In quite a few cases, the result description, and the demonatration/argument for the conclusion, reflecting the analysis, is very rough, not in sufficient detail, for example (but not limited to these examples):

1. line#68-69, “several epithelial cell markers”, what are these markers? Why these markers?

*Response: We thank the reviewer for the helpful suggestion. To further verify the intestinal epithelial cell identity, we performed immunofluorescent staining on in vitro cultured organoids with EPCAM, VIL and CDX2. EPCAM is a canonical epithelial cell marker, while VIL and CDX2 are well-known intestinal epithelial cell markers. Following the reviewer’s suggestion, we added references and detailed descriptions of these genes in corresponding figure legend and supplementary table (line 71-72, 678-680 and Supplementary table 3 in our revised manuscript, Table R1).*

*
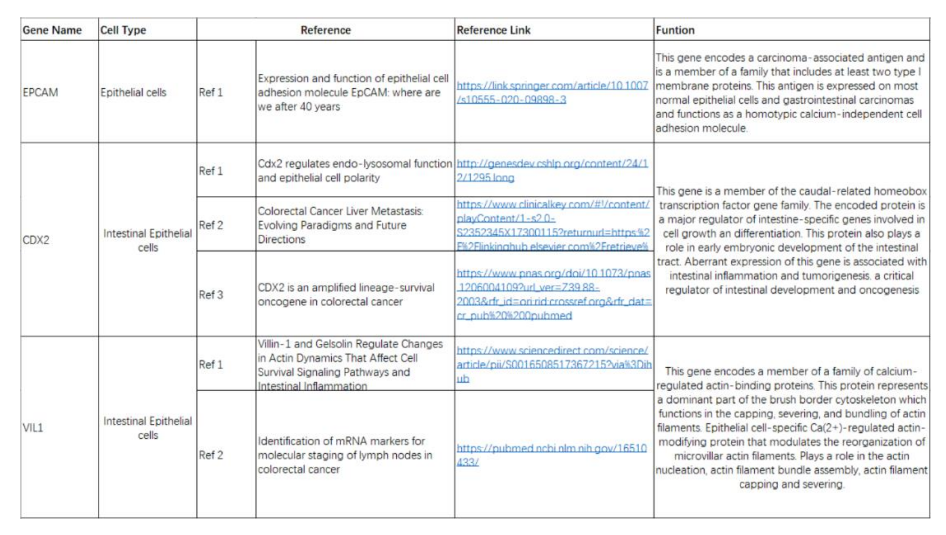
*

*Table R1. The table shows information of used gene markers in this manuscript.*

1. Line#86, annotated as “epithelial cells, immune cells and mesenchymal cells”, this classification seems too general. With more detailed clustering, some difference between conditions may become visible, especially the epithelial cells.

*Response: We thank the reviewer for the insightful comments and suggestions. Accordingly, in our revised manuscript, we used canonical intestinal cell type markers to further divide epithelial cells into seven subtypes (Figure R2-R3). Globally, we have found that compared with normal tissues or normal tissue-derived organoids, in vivo tumor cells and in vitro tumor-derived organoids have more stem cell-like cells. In addition, in vitro culture increases the ratio of stem cell-like cells in normal-derived organoid cells, which is also consistent with our conclusion that in vitro culture makes normal epithelial cells show tumor-like characteristics.*


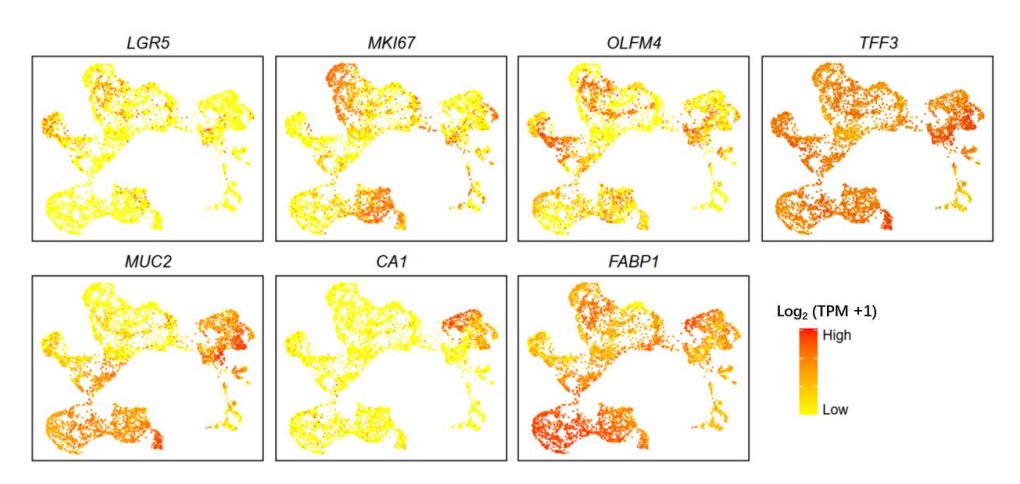


*Figure R2 Expression patterns of intestinal cell type markers were projected onto the UMAP plot. The colors from yellow to red represent expression levels from low to high. OLFM4 and LGR5, intestinal stem/pluripotent cell markers; MKI67, cell proliferative marker; CA1 and FABP1, enterocyte markers; MUC2 and TFF3, goblet cell markers.*


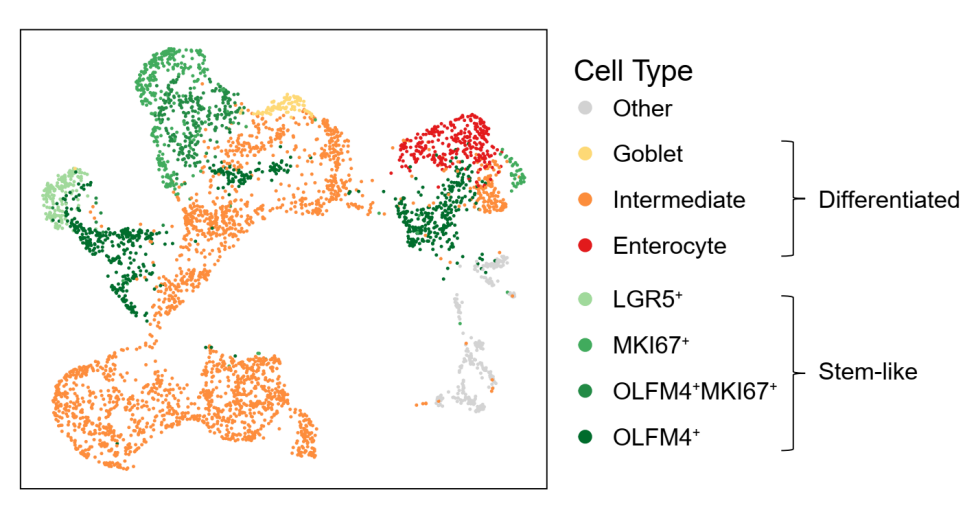


*Figure R3 UMAP clustering of cells and colors represent different epithelial subtypes.*

1. Line#86, furthermore, on the Right side of the FigS2B, it shows different pattern (subset of cells) between the In vivo tumor and the organoid tumor. How do the author interpret it?

*Response: We thank the reviewer for the insightful comments. Combined with the questions raised by the reviewers, we guessed that the reviewer wanted to ask about the clustering map in FigS2A instead of FigS2B. Sorry if the guess is wrong (Figure R4).*

*The reasons why tumor cells in vivo and in vitro cultured tumor organoids show different patterns, we guess it is mainly caused by the following two aspects. Firstly, it is due to the different growth environments in vivo and in vitro, such as the different growth factor concentration and the air condition (oxygen and carbon dioxide concentrations).*

*Secondly, it may be due to the lack of tumor microenvironment cells in the in vitro organoid culture system. Although the in vitro 3D culture model has simulated the three-dimensional structure between cells in vivo as much as possible, it still lacks the tumor microenvironment cells, since the in vitro culture medium is mainly suitable for the growth of epithelial cells but not for other cell types such as immune cells and stromal cells. For the tumor cells of some patients, if the influence of the in vivo microenvironment is relatively limited, it is possible that the tumor cultured in vitro can simulate the characteristics of the in vivo tumor to the greatest extent, so the differences between the in vivo tumor cells and in vitro tumor-derived organoids will be relatively small.*

*However, this speculation requires measuring the composition of the in vivo tumor microenvironments of different patients, and since we only sequenced a relatively small number of in vivo tumor cells for each patient, this speculation needs further data validation in the future.*


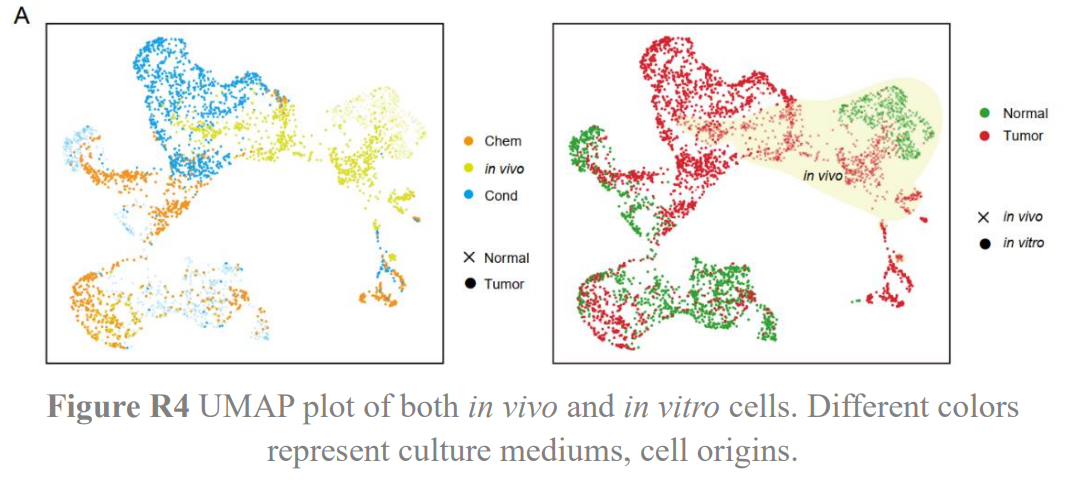


4) .line #100, which genes are important? Why significant and make sense in biology?

*Response: We thank the reviewer for the helpful suggestions. We have already highlighted the important and biologically significant genes in our revised manuscript. Moreover, we also added associated references in the supplementary tables accordingly.*

*It showed that several well-known intestinal marker genes (such as CLCA1, PYY, GCG, CA1, CLAN8 and SI) were highly expressed by both normal epithelial cells in vivo and normal tissue-derived organoid epithelial cells in vitro, which reflect that our normal tissue-derived organoids maintained the characteristic transcription features as well as epithelial cell sub-types of in vivo intestinal tissue (Supplementary Table 3 in the revised manuscript, Table R2). In addition, patient-derived tumor organoid cells in vitro also highly expressed CRC associated markers and many of the genes that are highly expressed in tumors have been widely reported to be closely related to colorectal progression and metastasis (Supplementary Table 3 in the revised manuscript, Table R3). Notably, normal tissue-derived organoids in vitro also expressed some of tumor specific genes. Taken together, these results indicated that tumor-derived organoids in vitro maintain the tumor cell features in vivo, while normal tissue-derived organoids only partially maintained some of the expression characteristics of normal tissues in vivo, and acquired some of tumor-like characteristics.*


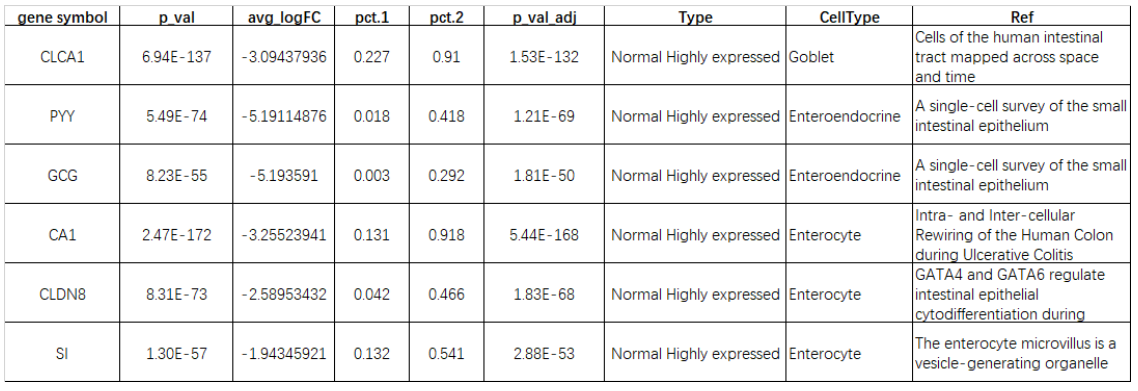


*Table R2 The table shows intestinal cell type markers that highly expressed by normal cells and their references.*


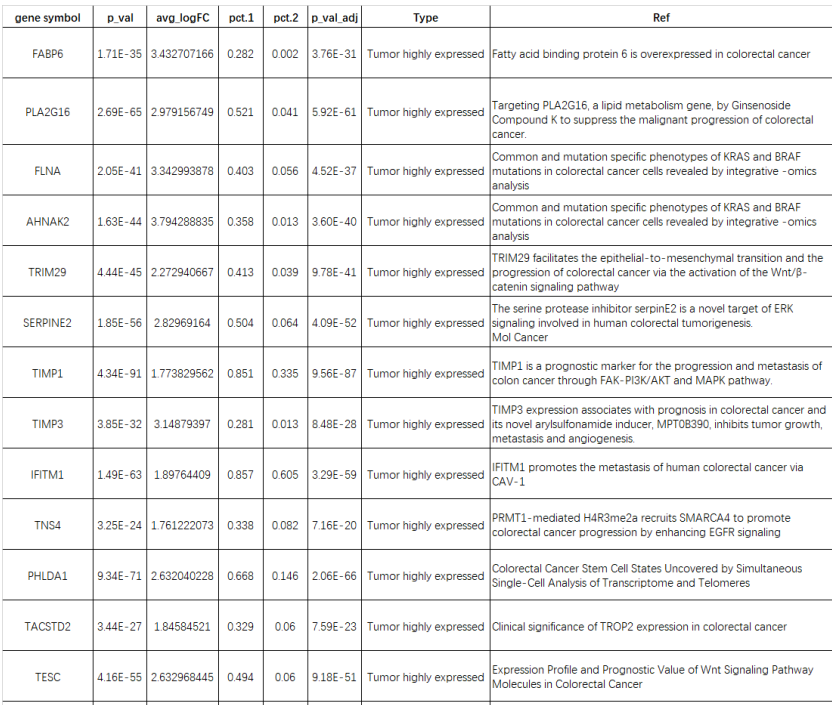

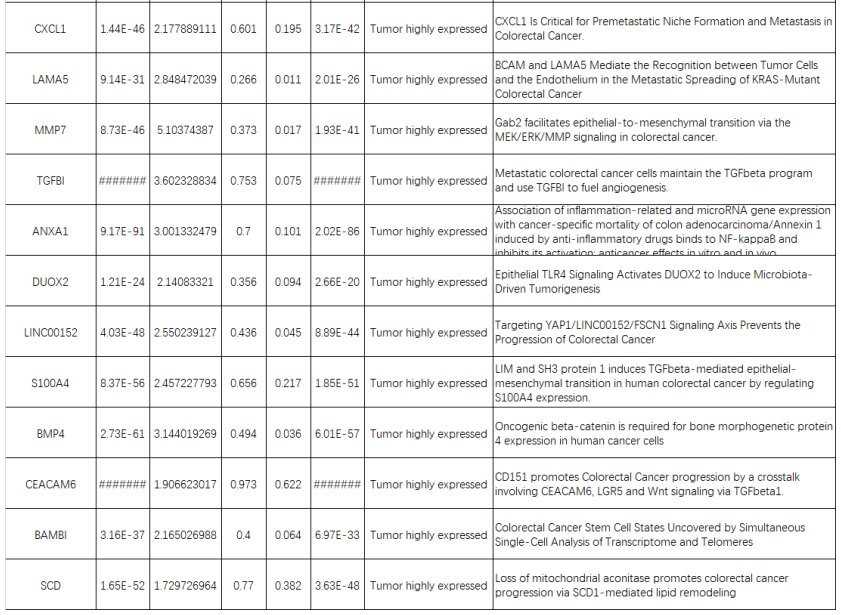


*Table R3 The table shows tumor highly expressed genes and their associated functions in cancer.*

5) Line#189-190, the result Fig 4F as well as the conclusion (therefore…) are not well interpreted or demonstrated.

*Response: We thank the reviewer for the comments. We have rewritten correspondence sentences in our revised manuscript (Line 196-203, 207-212). It is reported that cancer cells would exhibit partial EMT characteristics in order to enhance their invasive properties, generate circulating tumor cells and cancer stem cells, and promote resistance to anti-cancer drugs. When cancer cells showed partial-EMT state, it would simultaneously exhibit both mesenchymal and epithelial cell characteristics. VIM is a well-known mesenchymal cell marker and our data showed that tumor-derived organoid epithelial cells in vitro expressed both EPCAM and VIM simultaneously in the same individual cells, which indicated that the in vitro culture condition might favor the partial EMT features of tumor cells in organoids.*

6) Line#199, How is it verified? Where is the data?

*Response: Sorry for not describing it clearly. The data can be found in Figure S6-S7 and corresponding raw sequencing data have been uploaded to GSA database. As for WGS data, we performed single cell level and/or bulk level WGS sequencing for P1 and P8, which showed that tumor organoids in vitro maintained the CNV features of tumor cells in vivo, while the genomic features of normal organoids were also maintained normal without CNVs. In addition, through Sanger sequencing we further verified that only tumor organoid cells maintained the in vivo tumor specific mutations, and corresponding results were shown in Figure S6A and S7C (Figure R5-R7). Following the suggestion of the reviewer, we have rewritten the corresponding sentences to make them more accurate. Thanks the reviewer again for the helpful suggestions.*


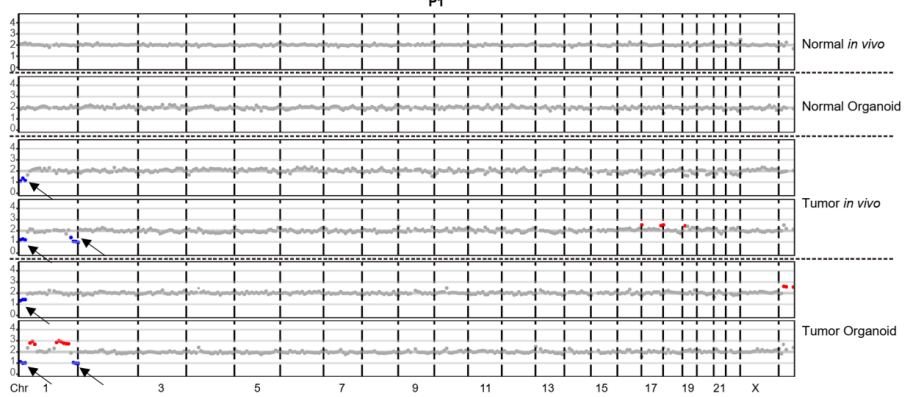


*Figure R5 The dot plot showing the CNVs pattern of Patient #1 that inferred. by whole genome sequencing data. Red, amplification; Blue, deletion. Grey, diploid.*


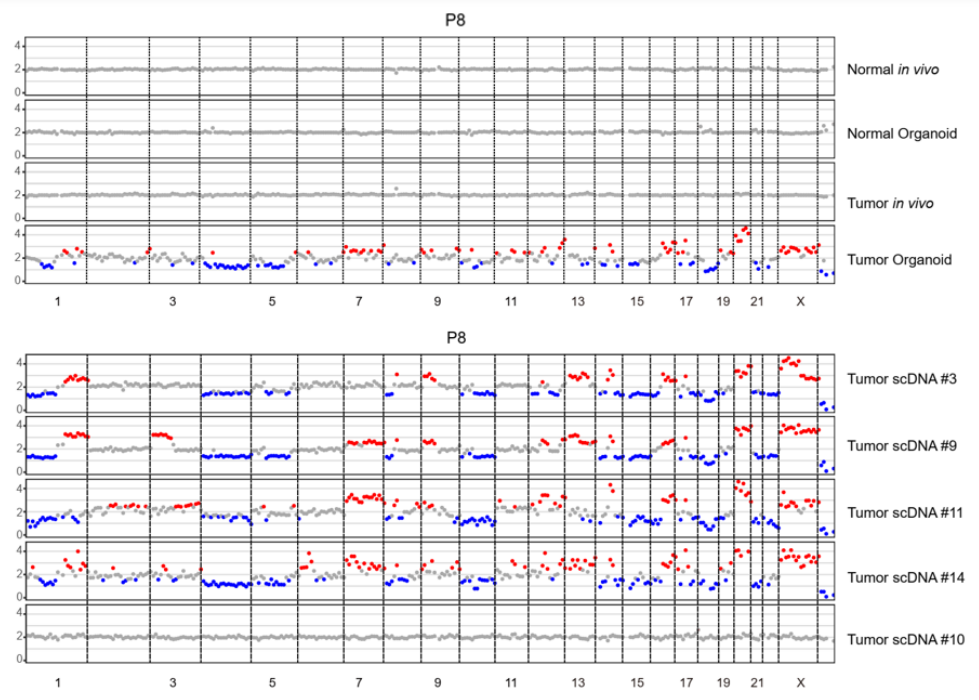


*Figure R6 CNV patterns of in vivo tissues and in vitro organoids that inferred by whole genome sequencing data for Patient #8. Red, amplification; Blue, deletion. Grey, diploid. Upper panel, bulk whole genome sequencing. Lower panel, single cell whole genome sequencing.*


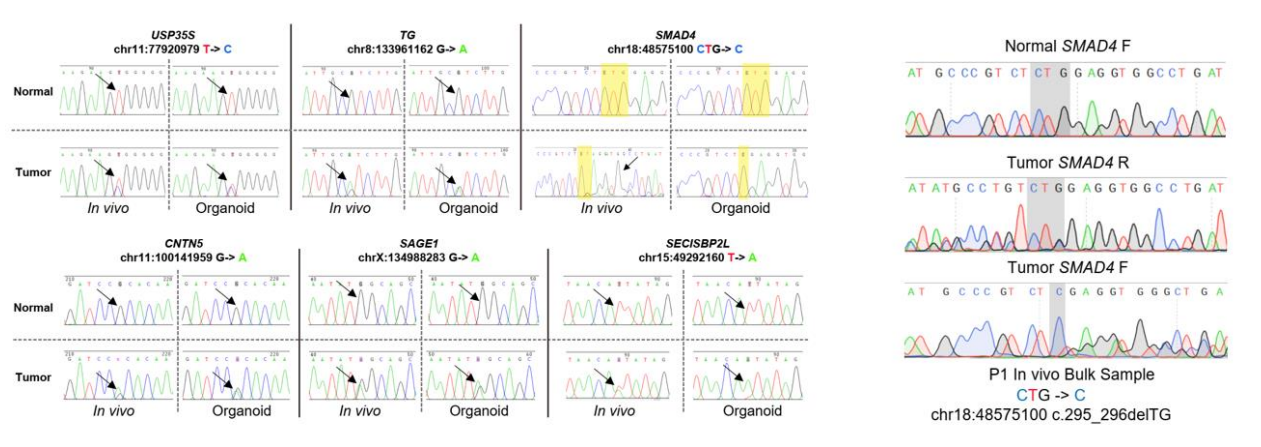


*Figure R7 Sanger sequencing results of tumor specific mutations*

7) Line#244, “due to lacking cell-cell interaction”, is it true?

*Response: We thank the reviewer for the comments, and we have rewritten this sentence to make the description more appropriate (Line 272-273).*

8) Line#563-564, Fig1(E): none of these markers is described for the function or significance.

*Response: We thank the reviewer for the suggestions. These markers are well-known markers for epithelial cells, mesenchymal cells and immune cells. We have added the descriptions of these genes in figure legends accordingly (Line 685-686).*

9) Line#579, Fig2(E): Is there any biological significance for these genes listed? Particularly the red genes on the right side?（figure 2A）

*Response: We thank the reviewer for the suggestions. We have added descriptions of these genes in our revised manuscript and summarized them in the supplementary table 3. Many of the tumor highly and specifically expressed genes were reported to play important roles in CRC progression and metastasis, and some of the normal tissue highly expressed genes were well-known intestinal cell type specific marker genes.*


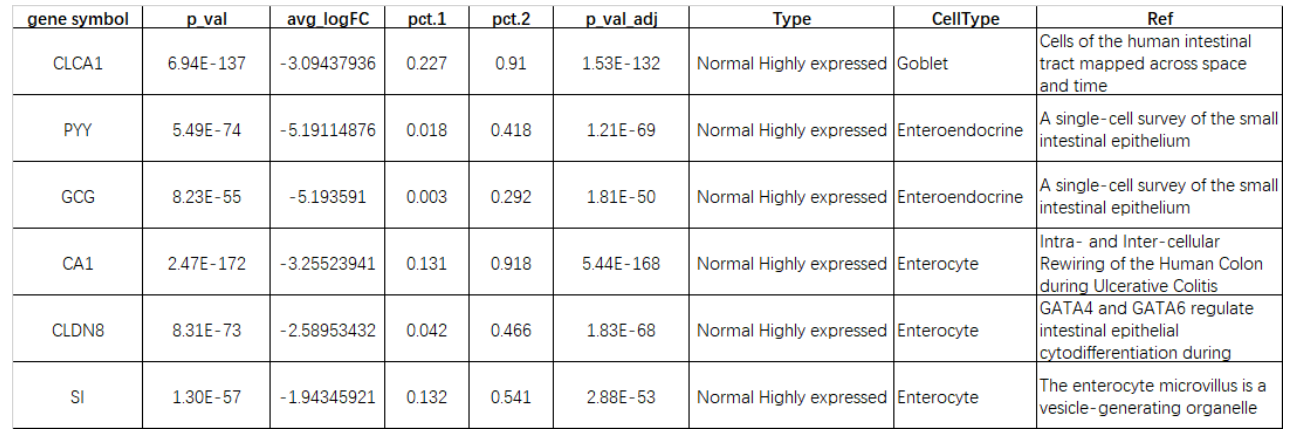


*Table R4 The table shows intestinal cell type markers that highly expressed by normal cells and their references.*

10) Line#672, FigS4: which and where are the 3 clones, or 4 clones?

*Response: Sorry for not describing it clearly. We have revised the figures and labeled the clone information next to the figures. We clustered the cells based on their CNVs patterns, and we defined cells possessing different CNVs as distinct clones (Figure S4 in the revised manuscript and Figure R8).*

11) Line#703, FigS7E: What do these data tell? Why these data are shown??

*Response: TSS and TES represent the transcription start site (TSS) and transcription end site (TES). The x axis represents the relative position of genes and the y axis represents the average DNA methylation levels. This figure reflects the global DNA methylation levels across the gene bodies. This figure reflects that the organoids cultured in vitro maintained the global DNA methylation signatures of the corresponding cells in vivo. In vivo normal tissues and in vitro normal tissue-derived organoids have similar DNA methylation profiles, and the methylation levels are higher than those of both in vivo tumor cells and in vitro tumor-derived organoids as expected.*


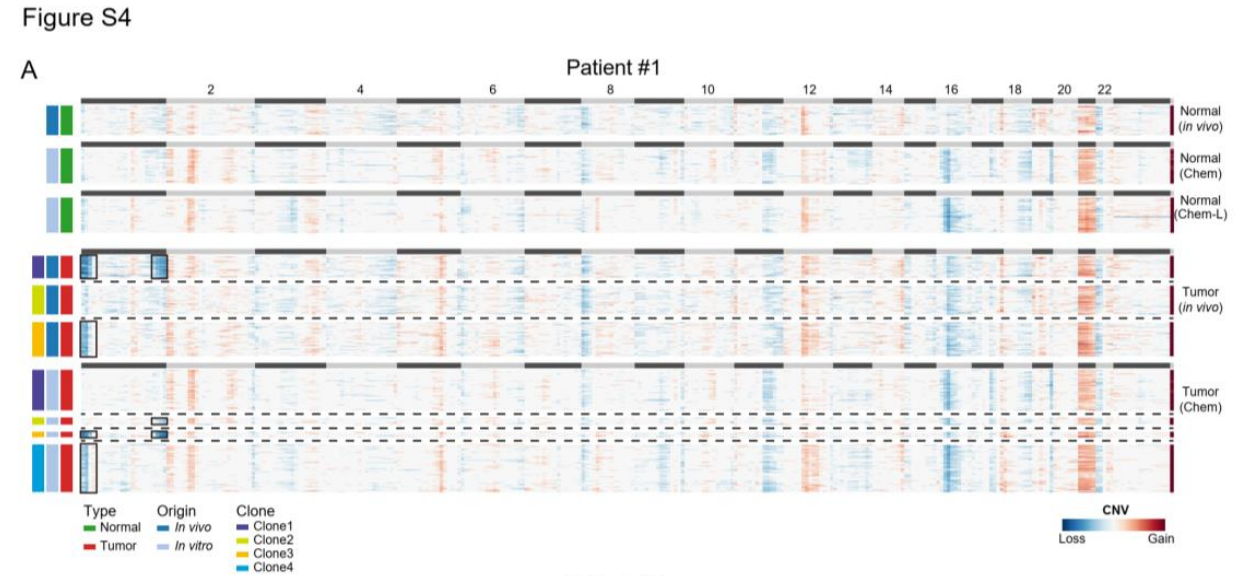

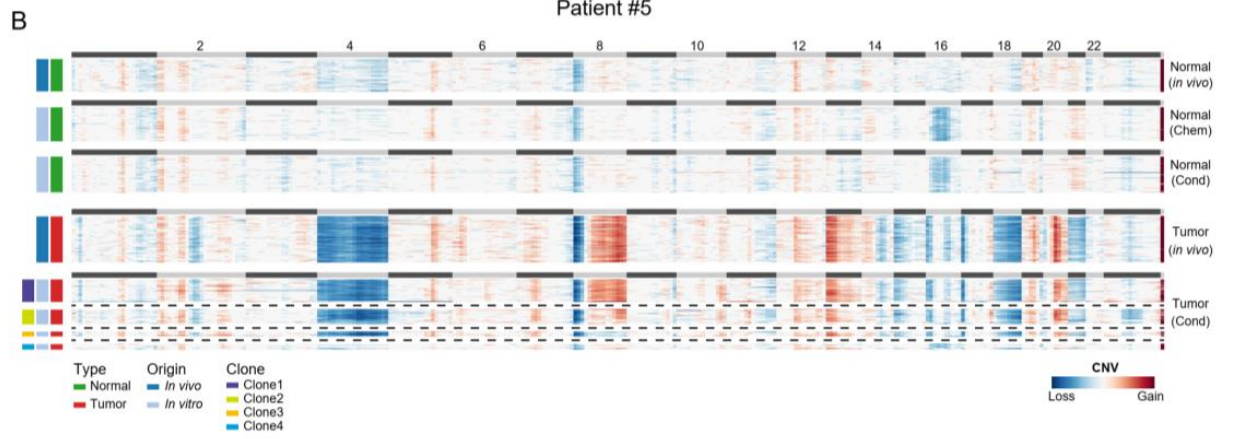

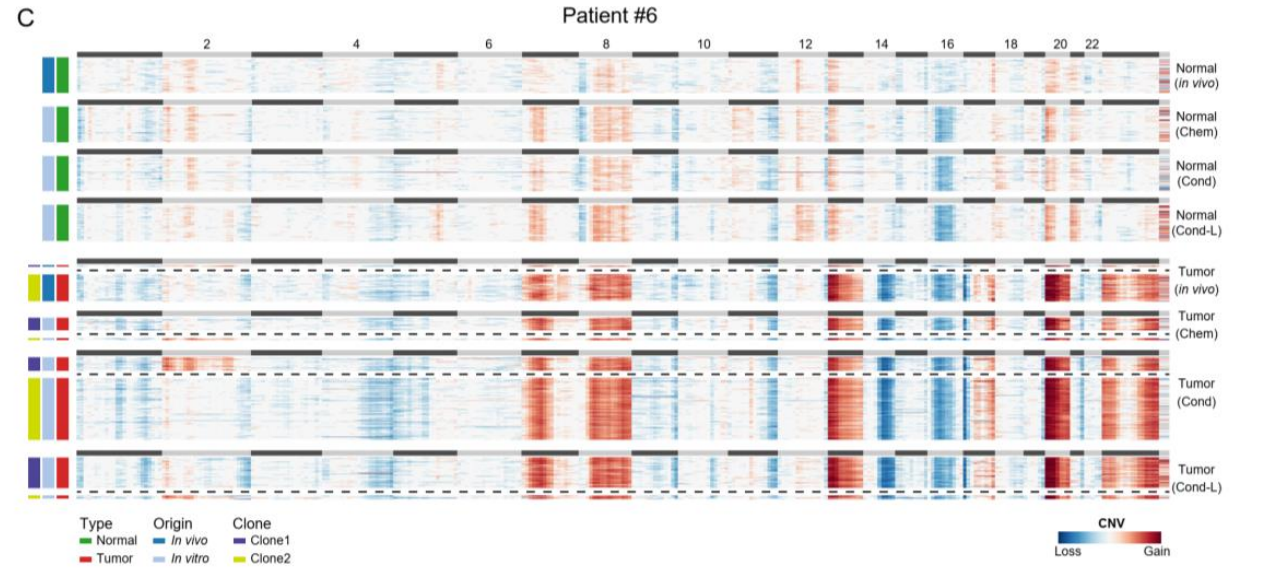


*Figure R8 The heatmap shows the CNV pattern of each cell with normal cells as control. Blue represents CNV deletion, while read represents CNV duplication.*

11) Line#703, FigS7E: What do these data tell? Why these data are shown??

*Response: TSS and TES represent the transcription start site (TSS) and transcription end site (TES). The x axis represents the relative position of genes and the y axis represents the average DNA methylation levels. This figure reflects the global DNA methylation levels across the gene bodies. This figure reflects that the organoids cultured in vitro maintained the global DNA methylation signatures of the corresponding cells in vivo. In vivo normal tissues and in vitro normal tissue-derived organoids have similar DNA methylation profiles, and the methylation levels are higher than those of both in vivo tumor cells and in vitro tumor-derived organoids as expected.*


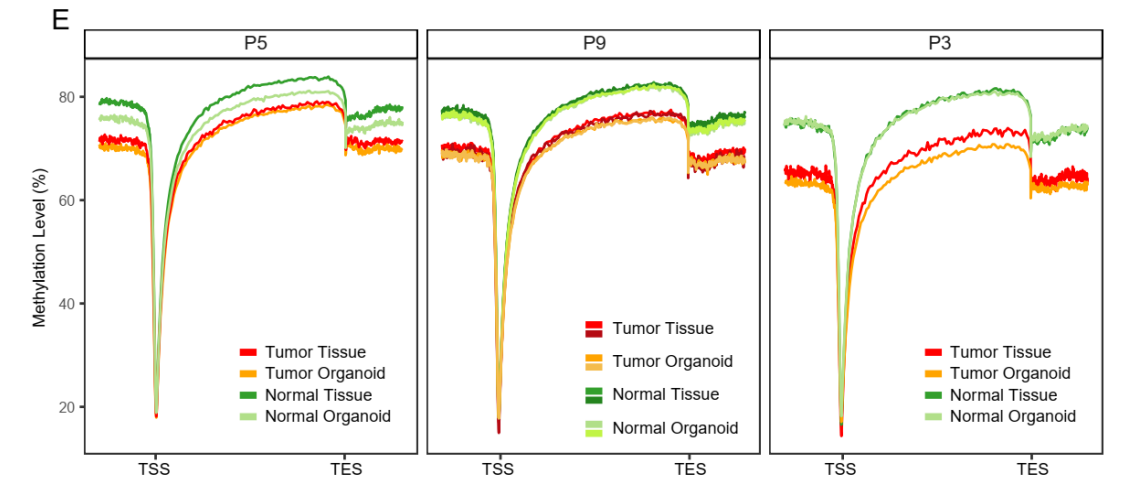


*Figure R9 Line plot showing the DNA methylation ration around transcriptional start site (TSS), gene body and transcriptional end site (TES). Different color represents cells in different regions.*

12) The paragraph started at line#216, with the DNA methylation, only a general profile is given, which is truly informative. However, is there any particular change for the normal tissue between the in vivo biopsy and the organoid? how about the answer of the tumor biopsy?

*Response: We thank the reviewer for the comments. Here, we only refer to changes in the global DNA methylation levels, and do not refer to the DNA methylation levels of specific genomic regions. In addition, following the reviewer suggestion, we calculated the methylation levels of different regulatory elements, it showed that except CGI, normal tissues and normal tissue-derived organoids have higher methylation levels in other regulatory elements compared with tumor tissues and tumor-derived organoids. However, since our sequencing depth is relatively shallow (sequencing to very high depth is very expensive), which is not enough for performing statistically test, to make the language more rigorous, we have toned down and revised the corresponding sentences (Line 239-249 in the revised manuscript) accordingly. We are very grateful to the reviewer for the constructive suggestions.*


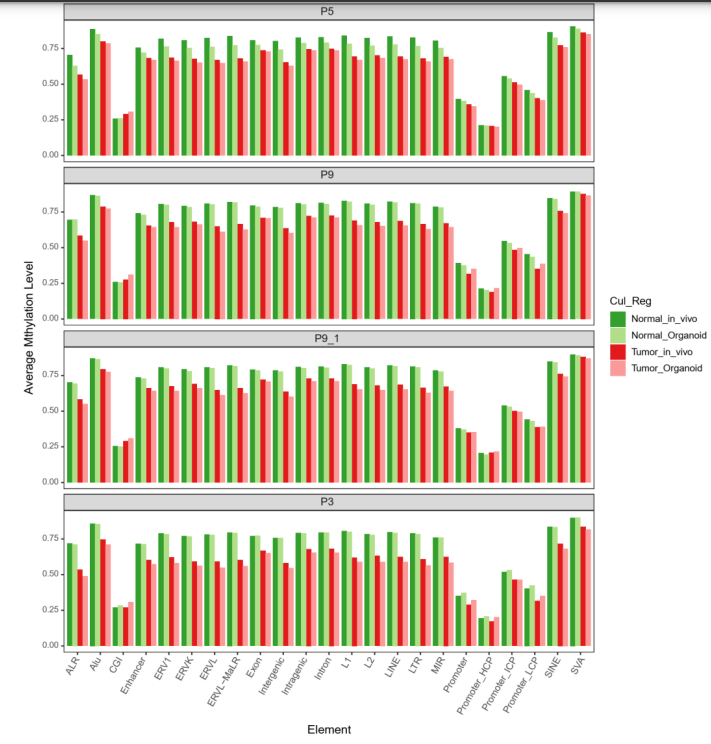


*Figure R10 Bar plot showing the average methylation levels of different regulatory elements.*

13) In the Abstract, regarding genomic (including eigenomic) profiling, the description does not give any detailed message, such as CNV or SNV mutation, or DNA methylation. This way a reader would not know better what you refer to

*Response: We thank the reviewer for the helpful suggestions. Following the reviewer’s suggestion, we have revised our abstract by adding more detailed descriptions accordingly.*

Minor Concerns: 1, About the technology 1) For single cell CNV calling, 2 methods are used for the set of same set of samples studied, scRNA-seq-inferred CNV, and MALBAC analyzed CNV. What are the correlation, consistence or inconsistence between these 2 methods?

*Response: We performed both single-cell RNA-seq (Figure R11) and single-cell whole genome sequencing (MALBAC) (Figure R12) for Patient #1. It showed that CNVs inferred by both single-cell RNA-seq data and single-cell WGS data are consistent with each other*


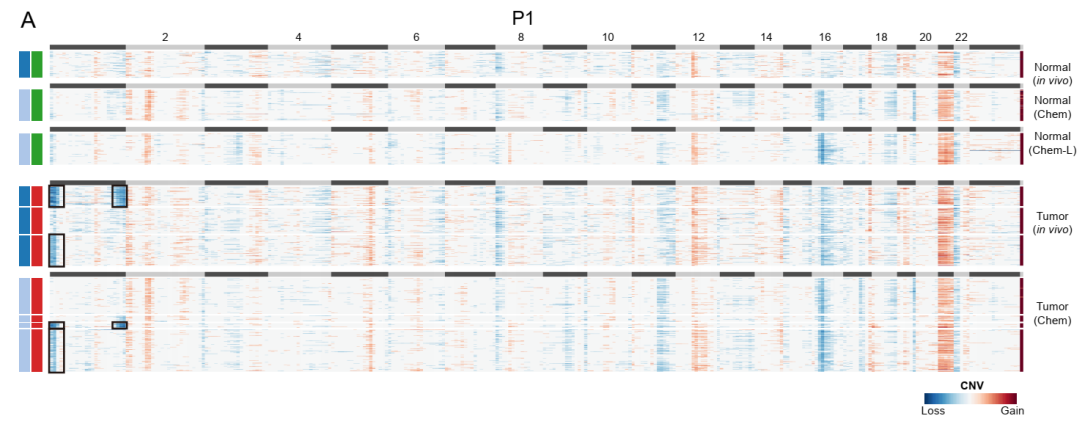


*Figure R11 Heatmap showing the CNV patterns of Patient #1 that inferred by single-cell RNA-seq data*


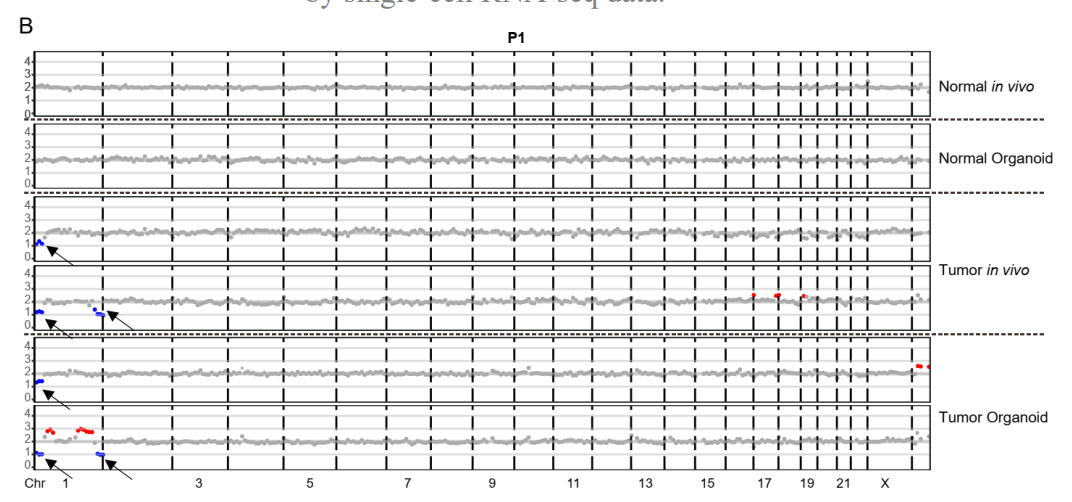


*Figure R12 Heatmap showing the CNV patterns of Patient #1 that inferred by single-cell WGS data.*

*2) For scRNA-seq, STRT method is used, so not very many cells are obtained with single cell data. What are the key advantages of STRT that the authors took so as to use STRT instead of 10x genomics or BD Rhapsody in this project?*

*Response: We thank the reviewer for the comments. The reason why we use modified STRT method instead of other high throughput method are as following: First, in order to explore the individual organoid’s origin, we picked single cells after dissociating a single organoid. Each organoid consists of just hundreds of cells, and some of them even only have dozens of cells. 10x genomics requires tens of thousands of cells for each run, so it is not possible for capturing cells from an individual organoid. Secondly, STRT method has higher sensitivity, can detect more genes per cell, and has lower dropout rates according to existing researches1,2 . So we choose STRT method in order to get high-quality single-cell sequencing data.*

*References： 1. Comparative Analysis of Single-Cell RNA Sequencing Methods 2. Benchmarking single-cell RNA-sequencing protocols for cell atlas project*

2, About spelling 1) Line#243, “… cell line which can not …” may be better to be” cell line, which can’t ” (with a comma “,” before which”) 2) Line#610, Is it correct “bottom left”? 3) Fig4H, “enrich for epithelial cell”, or “enrich for epithelial cells” (cell with “s” or without “s”)

*Response: We thank the reviewer for pointing these out. We have corrected them in our revised manuscript accordingly.*

**Reviewer 2**

**Reviewer #2**: In this manuscript, Wang and colleagues report a comparative genomic analysis of tumor and adjacent normal tissue-derived 3D organoids from patients with colorectal cancer. Using scRNA-seq, bulk and single-cell whole genome sequencing, whole exome sequencing (WES), whole genome bisulfite sequencing and Sanger sequencing, organoids were compared with each other, with their in vivo counterparts, and across distinct culture conditions. In doing so, the authors identified negligible genomic differences between tumor-derived organoids and paired in vivo tumor samples. In contrast, adjacent normal tissue-derived organoids exhibited some transcriptional hallmarks of primary tumor samples regardless of culture medium, yet largely maintained normal genomes. Additionally, the authors found that (i) a conditioned culture medium was superior for long-term culture of tumor epithelial cells and that (ii) characteristics observed in the original culture medium were generally maintained after exchanging one culture medium for another.

Overall, this paper, while lacking in innovation, includes an important message for the field: tumor-derived organoids, but not adjacent normal tissue-derived organoids, tend to faithfully recapitulate in vivo genomic characteristics. Optimizing culture conditions to better preserve in vivo features of normal adjacent tissue-derived 3D organoids represents a critical unmet need.

Despite the strengths of this study, there are several critical shortcomings that should be addressed.

***Response:*** *We sincerely thank the reviewer for the positive comments on our manuscript and also for the very constructive suggestions on how to improve the manuscript. Based on the reviewer’s suggestions, we have added more detailed descriptions of the analysis process in the method section and attached associated codes. In addition, we also performed additional bioinformatic analyses to further identify subtypes of epithelial cells.*

Major comments:
1. The authors' analysis of cell composition by scRNA-seq is rather pedestrian, with only three major lineages identified (epithelial cells, immune cells and mesenchymal cells). I suggest the use of granular marker genes, unsupervised clustering, and/or reference guided annotation to delineate cellular composition at considerably higher resolution in order to precisely distinguish cellular heterogeneity within each of these three compartments. For example, both tumor and normal colon epithelial cells, as well as stromal and immune subsets, have been extensively characterized by previous scRNA-seq studies **(e.g., PMID 32451460),** with marker genes and reference profiles that could be applied here. Such data could also serve as an anchor for Seurat integration.

***Response:*** *We thank the reviewer for the insightful comments and suggestions. Accordingly, in our revised manuscript, we used known cell type markers to further divide epithelial cells into seven subtypes (Figure 2.1 and 2.2).* *Globally, we have found that compared with normal tissues or organoids derived from normal tissues, tumor regions or tumor-derived organoids have more stem cell-like cells (Figure 2.3). In addition, in vitro culture increases the ratio of stem cell-like cells in normal-derived organoid cells, which is also consistent with our conclusion that in vitro culture makes normal cells showing tumor-like characteristics.*


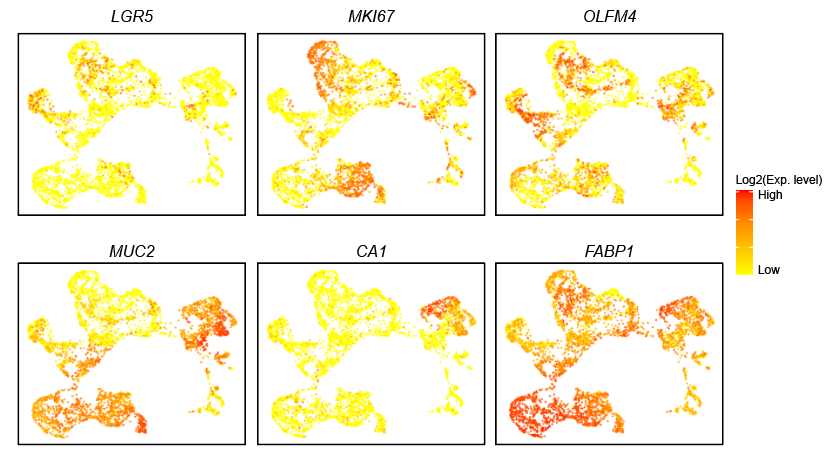


**Figure 2.1** UMAP plot showing the expression of well-known intestinal cell type markers. *LGR5* and *OLFM4*, intestinal stem cell marker; *MKI67*, proliferative marker; *MUC2*, goblet cell marker; *CA1* and *FABP1*, Enterocyte marker.


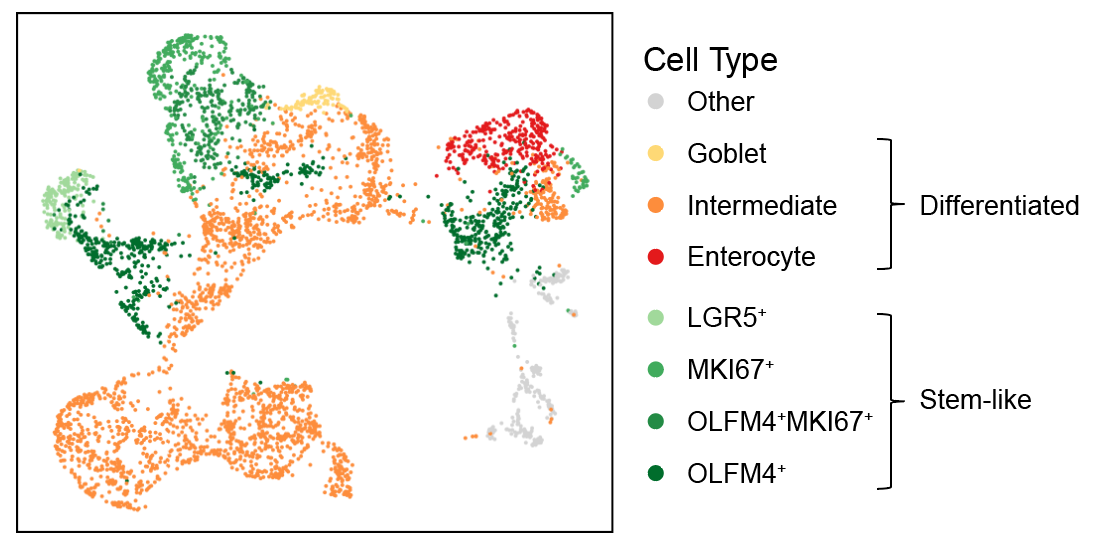


**Figure 2.2** UMAP plot showing epithelial subtypes. Other represent non-epithelial cells such as endothelial cells and immune cells.


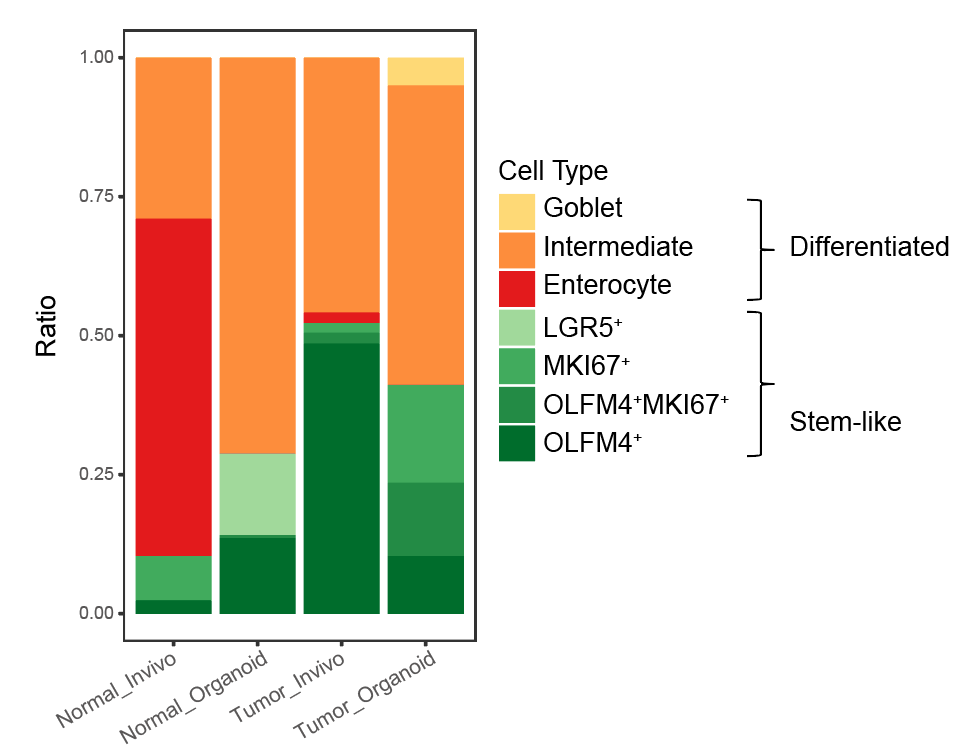


**Figure 2.3** Bar plot showing the ratio of each epithelial subtype in different cell origins.

2. Critical methodological details for scRNA-seq analysis, WGS analysis, WES analysis, and methylation analysis are missing. This is rather surprising given the focus of the paper. In general, references to previous literature ("as previously described") do not facilitate reproducibility. All methods, parameters used, software, and software version numbers should be provided in a self-contained manner. See related comments below.

***Response:*** *Thank the reviewer for pointing this out. In our revised manuscript, we added methodological details of the analysis process and attached a link to the corresponding codes.*

3. The network diagram in Figure 3A is cryptic. What tumor-specific regulatory network is shown? How was the regulatory network identified? Which genes are shown? Highlighting at least several key genes and providing a legend for the color scale would be helpful.

***Response:*** *We are sorry for not being clear enough. Each dot in Figure 3A represent an in vivo tumor highly expressed gene which identified by Seurat package FindMarkers function with setting ident.1= “Epithelial_Tumor_Invivo” and ident.2 = “Epithelial_Normal_Invivo” (other parameters set as following: logfc.threshold = 1.5,min.diff.pct = 0.25,min.pct = 0.25). The color of the plot represents the relative expression shown in the figure, and color from blue to red represent relative expression levels from low to high. For example, the color in first correlation network represents the relative expression between “tumor in vivo” and “normal in vivo” (which is equal to Mean (in vivo tumor cells)/ Mean (in vivo normal cells)). In details, we first calculated correlations of in vivo tumor-specific genes among in vivo tumor cells. Then, we used igraph package to construct the tumor-specific correlation network and removed vertices with less than three edges. Finally, we mapped the relative expression levels onto the network. Since these genes cannot be displayed clearly on the figure, so we put the gene list in Table 3. The analysis and code in this part are based on previously published article (Camp, J. et.al Nature, 2017). Following the reviewer’s suggestion, we added detailed descriptions in the Method Section.*

4. I suggest that the authors perform gene set enrichment and/or another pathway analysis method to more systematically explore transcriptional differences between organoid sources, organoid vs. in vivo tissues, and culture conditions.

***Response:*** *Thank the reviewer for the suggestion and comment. Following the reviewer’s suggestion, we performed GO and signaling pathway analysis with in vivo tumor and normal DEGs. Only FDR less than 0.05 was considerated as significant enrichment. We found that in vivo normal epithelial cells highly expressed genes associated with “negative regulation of growth” and genes involved in “mineral absorption”. As for in vivo tumor epithelial cells, there is no significantly enriched GO terms or KEGG pathway. These results can be found in Table 3.*


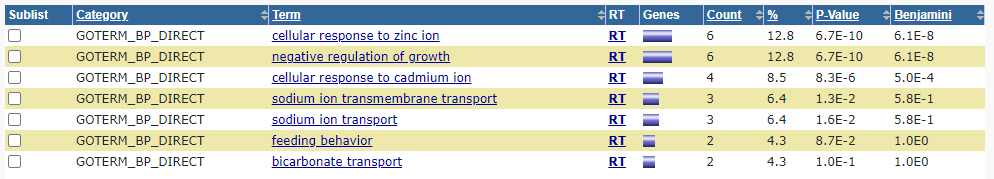


**Figure 2.4** In vivo normal cells highly expressed genes enriched GO terms.


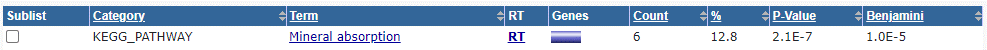


**Figure 2.5** In vivo normal cells highly expressed genes enriched KEGG pathways.


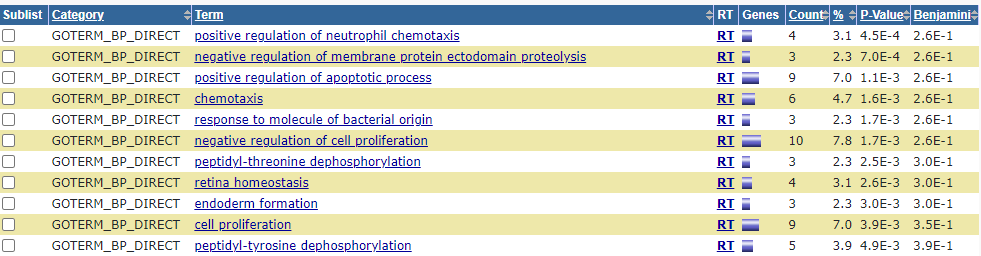


**Figure 2.6** In vivo tumor cells highly expressed genes enriched GO terms.


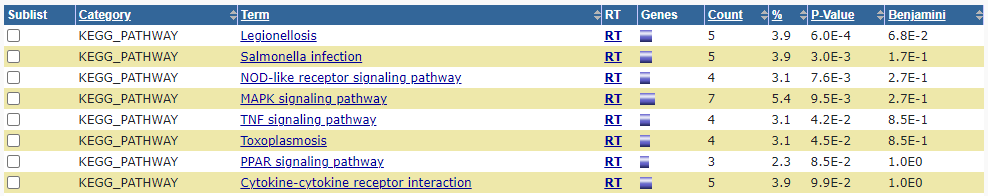


***Figure 2.7*** *In vivo normal cells highly expressed genes enriched KEGG pathways.*

*Then we also performed similar analysis on DEGs between in vivo and in vitro cells. It showed that there is no GO terms or pathways enriched for in vivo cell highly expressed genes. And in vitro cell highly expressed genes significantly enriched in ‘oxidation-reduction process’ GO terms. And it also highly expressed genes involved in digestion and epithelial cell differentiation, but does not statistically significant.*


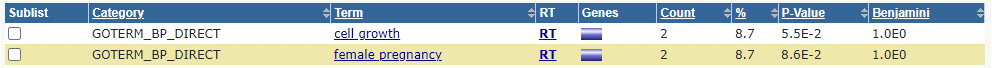


***Figure 2.8*** *In vivo cells highly expressed genes enriched GO terms.*

*
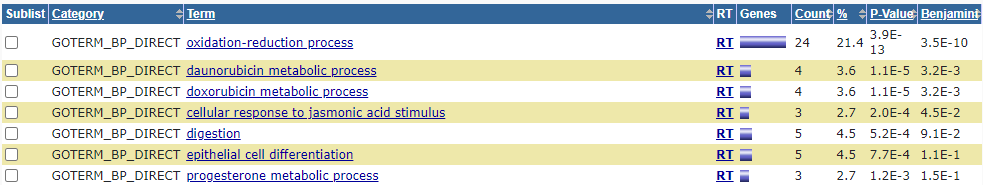
*

***Figure 2.9*** *In vitro cells highly expressed genes enriched GO terms.*

*
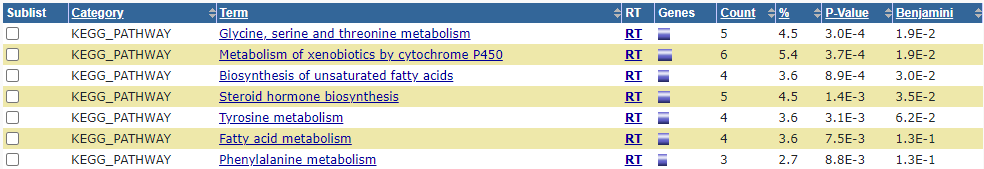
*

***Figure 2.10*** *In vitro cells highly expressed genes enriched KEGG pathways.*

*Finally, we performed GO and pathway enrichment for DEGs between cells cultured in different mediums. However, we did not found any significantly enriched GO terms or pathways.*


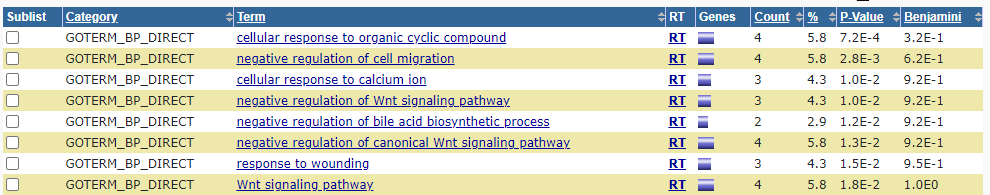


***Figure 2.11*** *Cells that cultured in chemical defined medium highly expressed genes enriched GO terms.*

*
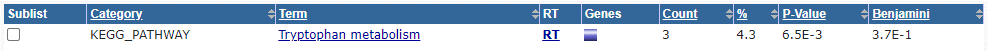
*

***Figure 2.13*** *Cells that cultured in chemical defined medium highly expressed genes enriched KEGG pathways.*

*
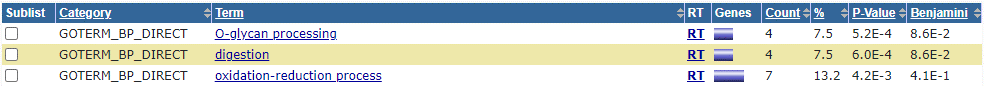
*

***Figure 2.14*** *Cells that cultured in conditional medium highly expressed genes enriched GO terms.*

*
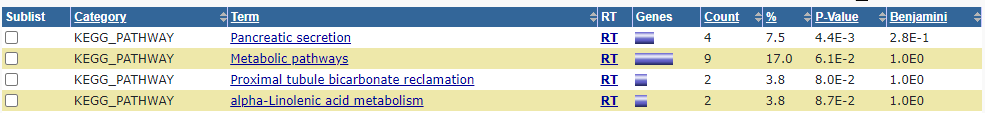
*

***Figure 2.15*** *Cells that cultured in conditional medium highly expressed genes enriched KEGG pathways.*

*Since we did not identify very interesting points during GO and KEGG pathway enrichment analysis, so we did not add this part of analysis in our revised manuscript. Thank the reviewer again for the suggestion.*

5. Details of DNA sequencing data are missing, for example the number of reads obtained per sample, the number/fraction properly paired and on-target, uniformity statistics for bulk/single-cell WGS, etc.

***Response:*** *We are sorry for not providing detailed descriptions. In our revised manuscript, we added these in Table 4. Thank the reviewer very much for pointing it out.*

6. The scRNA-seq preprocessing steps are unclear. Did the authors use % reads mapped to mitochondrial genes as a filtration criterion, as commonly applied to eliminate dead/dying cells? It appears not. The step involving the use of "cell-to-cell correlation (top 2 correlation higher than 0.6)" is odd; I assume this was done to remove doublets? No justification is provided for the approach or the threshold, and I wonder why the authors did not instead use an established doublet detection method, such as Scrublet.

***Response:*** *We did not use the percentage of reads mapped to mitochondrial genes as a filtration criterion. Because we used mouth pipette to collect single cell into the PCR tube, so we can judge the state of the cells and the doublet very easily through the microscope. And we only pick high-quality single cell for subsequent cDNA amplification and library constructions. The purpose of removing cells with low correlation is to remove outlier instead of removing doublets.*

7. Line 412: "Low-quality and index contaminate reads were first removed from the raw paired-end sequence data." What constitutes low quality?

***Response:*** *Bases with* *Q scores less than 38 were identified as low-quality bases and reads with more than 50% low-quality bases were identified as low-quality reads. The detailed code can be found in the GitHub website (s01.Barcode_UMI_QC_per1W_V2.pl at https://github.com/WRui/Post_Implantation/tree/master/scRNA_UMI/Split_Barcodes).*

8. Line 415: '10 M windows'. Windows of equal size?

***Response:*** *Yes, we divided the genome into equal size window (10M). The code to perform CNV analysis can be found in the GitHub (**https://github.com/WRui/Colon_FAP).*

9. Lines 416-417: Generally, copy number inference is performed against a paired germline control. By their methodological description, it is unclear whether the authors employed germline controls. The rationale for the read normalization approach is unclear and should be both justified and elaborated.

***Response:*** *Thank the reviewer for the comment and sorry for not making it clear. We have used three methods to infer the CNVs based on WGS data. In addition to just normalized read depth, we also used average read number of normal samples or all samples within each window as control. Since we also need to estimate the CNV of normal cells and existing papers have verified that normal cells might also contain a low ratio of cells that harbor CNVs (Li, R., et.al. Nature 2021; Zhou, Y., et.al. Cancer Cell 2021). If normal cells are used as a control to evaluate the copy number, it is assumed that all normal cells are diploid without CNVs, but in fact, normal cells may also have copy number variations, which will cause CNV prediction errors. Therefore, we did not use normal cells as a control, but just normalized the read number. In our revised manuscript, we have attached link of the corresponding codes.*

10. Lines 419-426: The entire WES pipeline should be specified in detail.

***Response:*** *Thank the reviewer for the insightful suggestion. We added analysis details and associated code link in our revised manuscript.*

11. How were mitochondrial mutations identified? What quality control steps were employed?

***Response:*** *We called the mitochondrial mutations using the output bam files of Tophat. Then, following the online GATK RNA-seq SNP calling tutorial, we called mutations on mitochondrial DNA. The SNP calling* *pipeline can be found at GitHub (https://github.com/WRui/Metastatic-Colorectal-Cancer). We have added the pipeline of mitochondrial mutation calling in the Method section in our revised manuscript.*

12. How was methylation calling performed?

***Response:*** *Sorry for not providing methodological details of methylation calling and we have added it in our revised manuscript.*

13. Line 310: "Organoid culture was performed as previously described". This is missing a reference and should ideally include a full description of the procedure here.

***Response:*** *We sincerely thanks the reviewer for reminding us. We have inserted the reference and made a complete description of the organoid culture procedure in our revised manuscript. The specific procedure was shown as below:*

*Organoid culture was performed as previously described (Van de Wetering M, et.al, Cell, 2015), with minor modifications to the tissue dissociation protocol to improve cell viability. Specifically, well-washed adjacent normal tissues were carefully cut into 1-3 mm3 small fragments by a scalpel. The muscle layer was then stripped off from mucosa layer. Subsequently, fragments were incubation in 5μM EDTA for 15min with vigorously shaking. The isolated crypts were collected and further washed twice with basal medium. Then, the crypts were allowed to mix with BME (Cultrex Reduced Growth Factor Basement Membrane Extract, Type 2, Bio-techne) and seeded in 24-well plates (50 μL BME/well). Culture medium was added after the BME was well solidified (approximately 20 min after seeding). Tumor tissues were cut into small pieces and digested by collagenase (type II and type IV; Invitrogen) for 30 min at 37°C with vigorous pipetting every 5 min. After digestion, dissociated tissues were passed through a 40 μm cell strainer (Corning). The cell suspension was first centrifuged at 400 g for 5 min at 4°C and then was re-suspended with Matrigel and seeded in 24-well plates (50 μL BME/well). The composition of organoid culture medium was as follows:*

*Chemical defined medium: Advanced DMEM/F12 (Gibco), 100 U/mL penicillin/streptomycin (Gibco), Primocin (InvivoGen), 2 mM GlutaMAX (Gibco), 0.5 μM A83-01 (Tocris), 1x B27 (Gibco), 5 μM SB202190 (Sigma), 100 nM prostaglandin E2 (Tocris), 0.5 μg/mL R-spondin (Peprotech), 4mM nicotinamide (Sigma), 10 nM gastrin I (Sigma), 50 ng/ml EGF (Peprotech), 100 ng/ml Noggin (Peprotech), 100 ng/mL WNT3A (Millipore), 10ng/ml FGF-10 (Peprotech), 10ng/ml FGF-basic (Peprotech). Notably, 10 μM Y-27632(Selleck) was supplemented to the medium in the first week. The medium was were changed every 2 days.*

*Conditional medium: 50% conditioned media (1:1 diluted with Advanced DMEM/F12), 100 U/mL penicillin/streptomycin, Primocin (InvivoGen), 2 mM GlutaMAX (Gibco), 0.5 μM A83-01 (Tocris), 1x B27 (Gibco), 5 μM SB202190 (Sigma), 100 nM prostaglandin E2 (Tocris), 4mM nicotinamide (Sigma), 10 nM gastrin I (Sigma), 50 ng/mL EGF (Peprotech), 10ng/mL FGF-10 (Peprotech), 10ng/ml FGF-basic (Peprotech). The conditioned media was prepared according to the protocol as described (Miyoshi H., et.al., Nat Protoc, 2013)*

*Basal medium: 45mL Advanced DMEM/F12 (Gibco), 5mL FBS (Vistech), 500 μL GlutaMAX (Gibco), 100 U/mL penicillin/streptomycin (Gibco), Primocin (InvivoGen)*

Minor comments:
1. UMAP in Figure 1D: This is a result of integration across samples, yet sample ID and culture conditions are relegated to Fig S1A. For coherence, I would suggest adding Fig S1A to Figure 1.

***Response:*** *According to the reviewer’s suggestion, we moved the original Figure S1A to Figure 1F.*

2. Figure 5 heat map: expression level values are not aligned with the color bar in the legend.
***Response:*** *Following the reviewer’s suggestion, we added the description of color bar in the figure legend.*

**Second round of review**

**Reviewer 1**

N/A

**Reviewer 2**

The revised manuscript by Wang and colleagues addresses several of my previous concerns. However, the following methodological aspects of this paper remain insufficiently explained or unconventional, and some clarifications made in response to my comments are inexplicably limited to the rebuttal:
1. Previous Major Comment #1: Please add a description to the Methods section detailing how cell type-specific marker genes were selected and used to delineate the clusters in Figure S2. Currently, the authors state that only three major cell types were annotated (lines 427-428).
2. Previous Major Comment #3: Add a colorbar to the figure.
3. Previous Major Comment #6: I am not convinced by this qualitative determination of quality. At a minimum, the authors should include a figure and analysis of % reads mapped to mitochondrial genes for every cell (as is convention in the field) and explain why they refuse to follow convention in the paper (typically cells with >25% reads mapped to mitochondrial genes are omitted). Furthermore, the cell-to-cell correlation approach "(top 2 correlation higher than 0.6)" remains insufficiently described and justified.
4. Previous Major Comment #7: Add details in response to Methods.
5. Previous Major Comment #8: Add details in response to Methods.
6. Previous Major Comment #9: The CNV inference approach used by the authors is both unconventional and flawed, as normal reference cells (Normal in vivo) show spurious amplifications and deletions after application of the authors' procedure, likely reflecting localized regions of coordinated gene expression (Figure S4). The authors' contention that "normal cells may also have copy number variations" is unlikely to be a confounding factor in practice. I suggest that the authors apply a dedicated scRNA-seq CNV inference method, such as CopyKat or inferCNV, to either augment or replace their current analysis, using normal in vivo cells as the reference.

**Authors Response**

**Point-by-point responses to the reviewers’ comments:**

The revised manuscript by Wang and colleagues addresses several of my previous concerns. However, the following methodological aspects of this paper remain insufficiently explained or unconventional, and some clarifications made in response to my comments are inexplicably limited to the rebuttal: 1.Previous Major Comment #1: Please add a description to the Methods section detailing how cell type-specific marker genes were selected and used to delineate the clusters in Figure S2. Currently, the authors state that only three major cell types were annotated (lines 427-428).

*Response: We thank the reviewer for the insightful suggestions. Following the reviewer’s suggestions, we rewrote the method sections in our revised manuscript. As for the three major cell types we mentioned in our manuscript we mean immune cells (PTPRC), epithelial cells (EPCAM) and mesenchymal cells (THY). Since epithelial cells were the focus of our study, we further classified epithelial cells into four subtypes. Specifically, MUC2 (Goblet), CA2 (Enterocyte) and FABP1(Enterocyte) were used to annotate differentiated epithelial cell types, whereas the cells with high expression of LGR5, MKI67 and OLFM4 were annotated as stem-like cell types. Many thanks for the reviewer’s suggestions. We have revised the description in this section in our revised manuscript accordingly. All of the markers we used are canonical cell-type specific marker genes that were widely used in the literature. We have added associated references of these makers in our revised manuscript (Supplementary Table 3; Revised Table 1).*


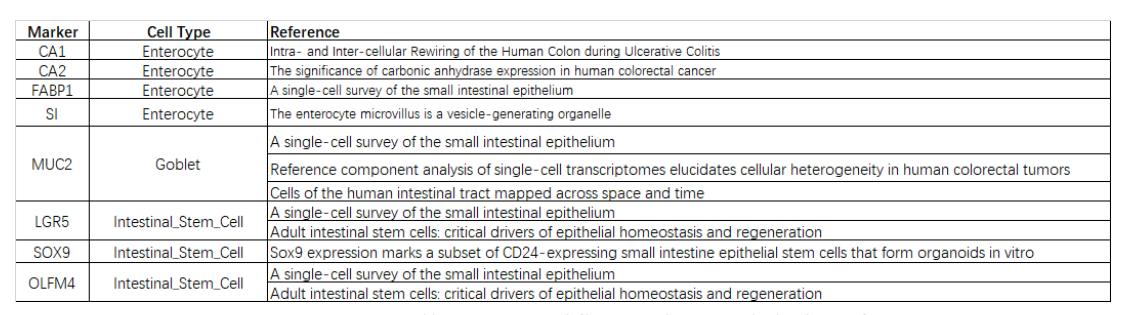


*Revised Table 1. Cell type specific marker and their references.*

2.Previous Major Comment #3: Add a colorbar to the figure.

*Response: We thank the reviewer for pointing it out. We have added color bar in our revised manuscript accordingly (Fig. 3A in the revised manuscript and Revised Figure 1).*


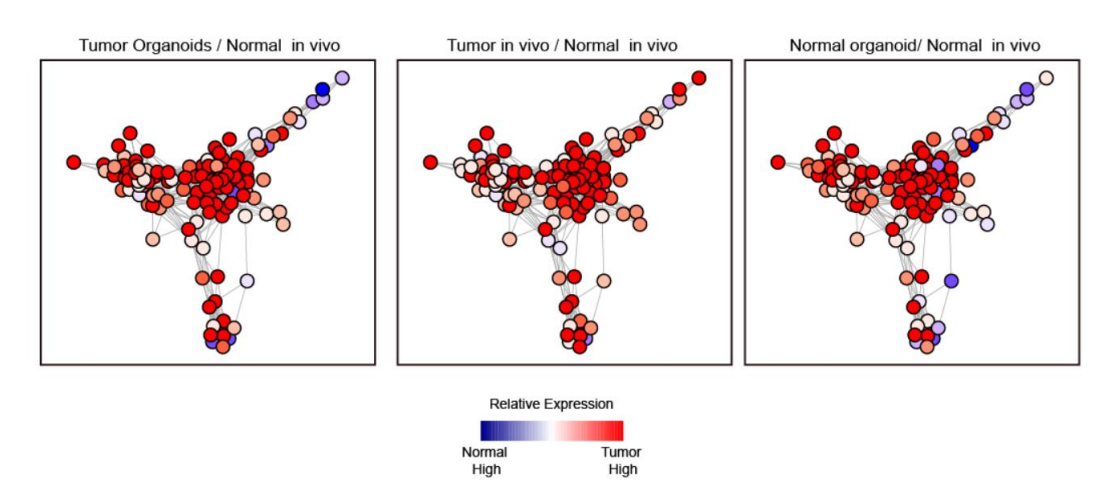


*Revised Figure 1 In vivo tumor-specific gene regulatory network. Nodes represent tumor-specific genes and their regulatory genes. The colors represent the fold change in the mean gene expression levels between the two sources of epithelial cells. (Left: tumor-derived organoid compared with normal tissue in vivo; right: normal-derived organoid compared with normal tissue in vivo)*

3.Previous Major Comment #6: I am not convinced by this qualitative determination of quality. At a minimum, the authors should include a figure and analysis of % reads mapped to mitochondrial genes for every cell (as is convention in the field) and explain why they refuse to follow convention in the paper (typically cells with >25% reads mapped to mitochondrial genes are omitted). Furthermore, the cell-to-cell correlation approach "(top 2 correlation higher than 0.6)" remains insufficiently described and justified.

*Response: We thank the reviewer for the comments. The median ratio of reads that mapped to mitochondria is 13.4%, which indicates the relatively high quality of our data. In addition, we also showed the ratio of reads mapped to mitochondria in organoids and in vivo tissues respectively, and found that the ratio is much lower in organoids than in vivo tissues, which further reflect the high quality of organoid samples (Revised Figure 2). In our manuscript, we used mutations in mitochondria to infer the clonal structure of organoids and in vivo tumor cells, and a little higher ratio of mitochondrial reads can make the clonal identification more accurate. In summary, by looking at the global mitochondrial ratio, it can be concluded that the quality of our data is reliable, and we want to use mitochondrial data for clonal analysis, so we do not use the mitochondrial ratio as a filter condition, but use the correlation coefficient between cells to filter out the outlier cells.*


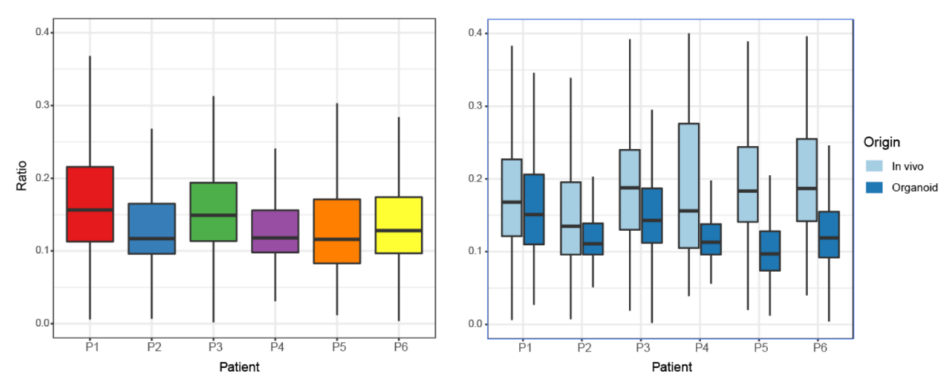


*Revised Figure 2 The box plot shows the ratio of reads mapped to mitochondria. Left, different colors represent different individuals. Right, different colors represent the cell origin.*

4.Previous Major Comment #7: Add details in response to Methods.

*Response: We thank the reviewer for the suggestions. We added the following descriptions in our revised manuscript accordingly (Line 433-440 in the revised manuscript). For each read R1, the polyA sequences were trimmed off, followed by filtering for high-quality reads with the following criteria: 1) at least 40bp long; 2) more than half of the bases showing the sequence quality scores greater than 38; 3) less than 10% of the bases showing N. In addition, the first 1-8bp and 9-16bp of Read2 were extracted as cell barcode and UMI sequence and added to the read ID. The detailed codes have already been uploaded to the GitHub (https://github.com/WRui/Post_Implantation/blob/master/scRNA_UMI/Split_Barcode s/s01.Barcode_UMI_QC_per1w_V2.pl), so the reader can get all details of the method.*

5.Previous Major Comment #8: Add details in response to Methods.

*Response: We thank the reviewer for the suggestion. We have added more details in the Method section of our revised manuscript accordingly (Line 493-506 in the revised manuscript). Control-FREEC (v11.3) was used to detect copy-number changes. Based on the tutorial (http://boevalab.inf.ethz.ch/FREEC/tutorial.html#CONFIG), the config file was created, which used the following setting parameters: chrLenFile=/Path/To/hg19.genome, ploidy = 2, window = 10000000, chrFiles=/Path/to/hg19_chrSeq, maxThreads= 2, BedGraphOutPut = True, samtools = /path/to/samtools, outputdir = /path/to/outdir, inputFormat = BAM, mateOrientation=0 and mateFile = /path/to/bamfile. Then we run ‘freec --conf config_file’ to get number of reads within per window (https://github.com/WRui/Colon_FAP/blob/master/04.FreeC_Work_Human.sh). Finally, we used R to normalize the total read depth and then use ‘ggplot2’ package to visualize the CNV patterns. The R script used to normalize read depth and data visualization can be found in the GitHub website (*[*https://github.com/WRui/Colon_FAP/06.Plot_CNV.sh*](https://github.com/WRui/Colon_FAP/06.Plot_CNV.sh)*).*

6.Previous Major Comment #9: The CNV inference approach used by the authors is both unconventional and flawed, as normal reference cells (Normal in vivo) show spurious amplifications and deletions after application of the authors' procedure, likely reflecting localized regions of coordinated gene expression (Figure S4). The authors' contention that "normal cells may also have copy number variations" is unlikely to be a confounding factor in practice. I suggest that the authors apply a dedicated scRNA-seq CNV inference method, such as CopyKat or inferCNV, to either augment or replace their current analysis, using normal in vivo cells as the reference.

*Response: We thank the author for the suggestions and sorry for not describe it clearly. The code we used was modified from inferCNV, and we just made minor changes to make it much easier to change the color setting and change clustering method. Even with inferCNV (https://www.bioconductor.org/packages/release/bioc/vignettes/infercnv/inst/doc/infer CNV.html ), some normal diploid cells will show light red or blue, which seems to have an increase or decrease in copy number. In fact, this is background noise, so only the very dark color Red and blue are considered as true copy number increases or decreases. So there is nothing wrong with the method we used to infer the CNVs.*


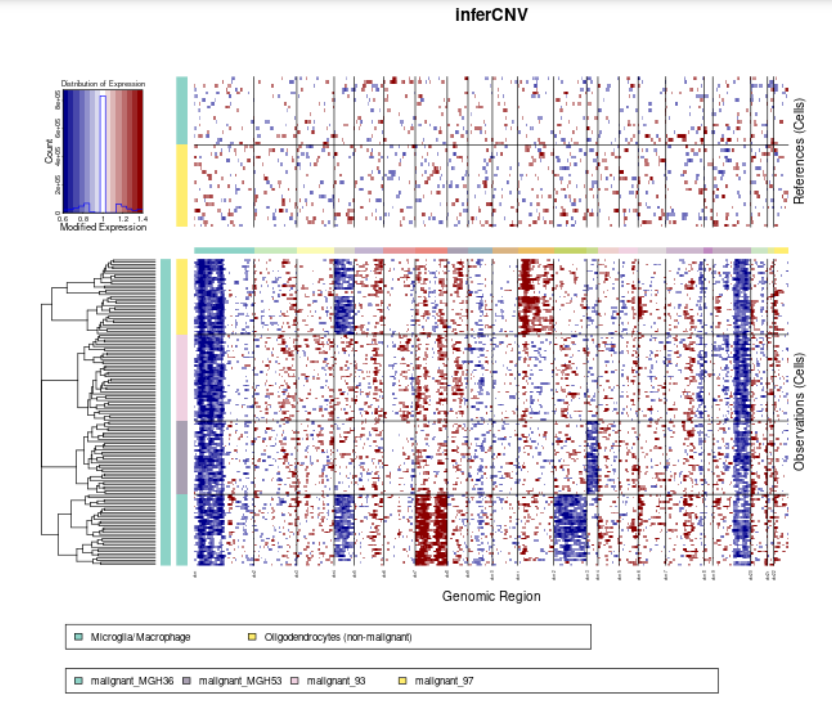


*Revised Figure 3 The heatmap showing the CNV patterns that inferred by inferCNV package and this figure This picture comes from the official tutorial website of inferCNV.*

**Third round of review**

**Reviewer 1**

My questions have been well addressed. I have no further question.

**Reviewer 2**

The authors have satisfactorily addressed my comments.
